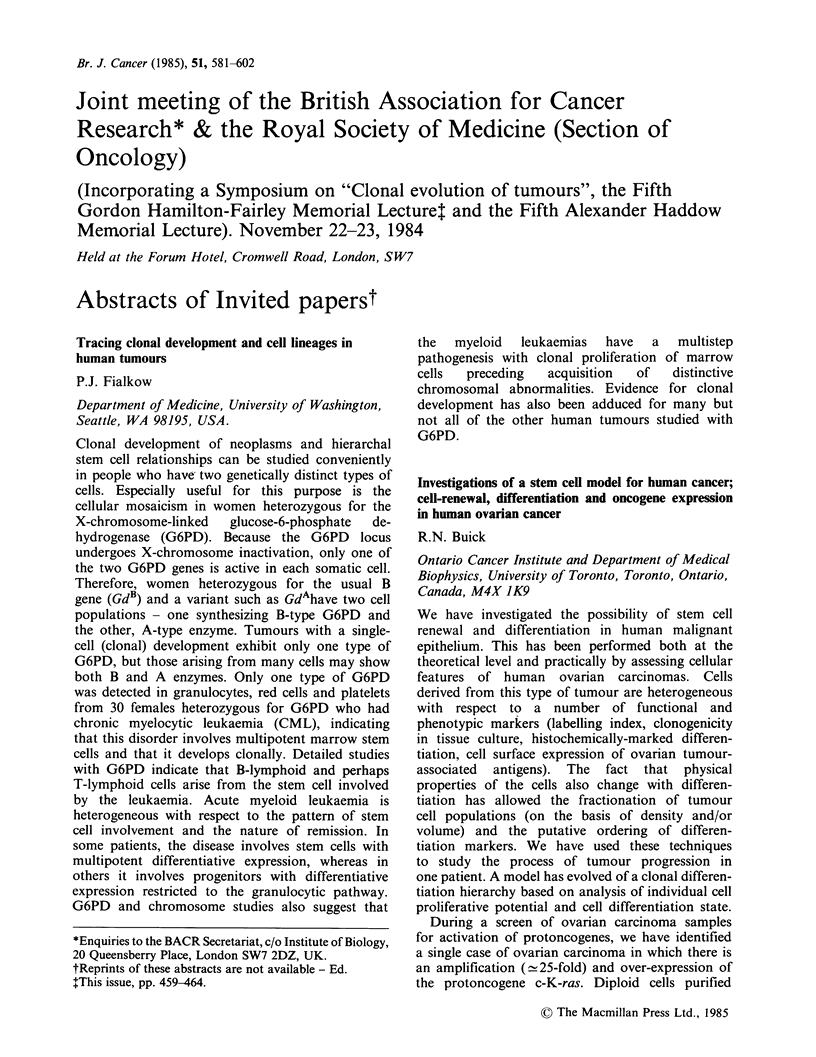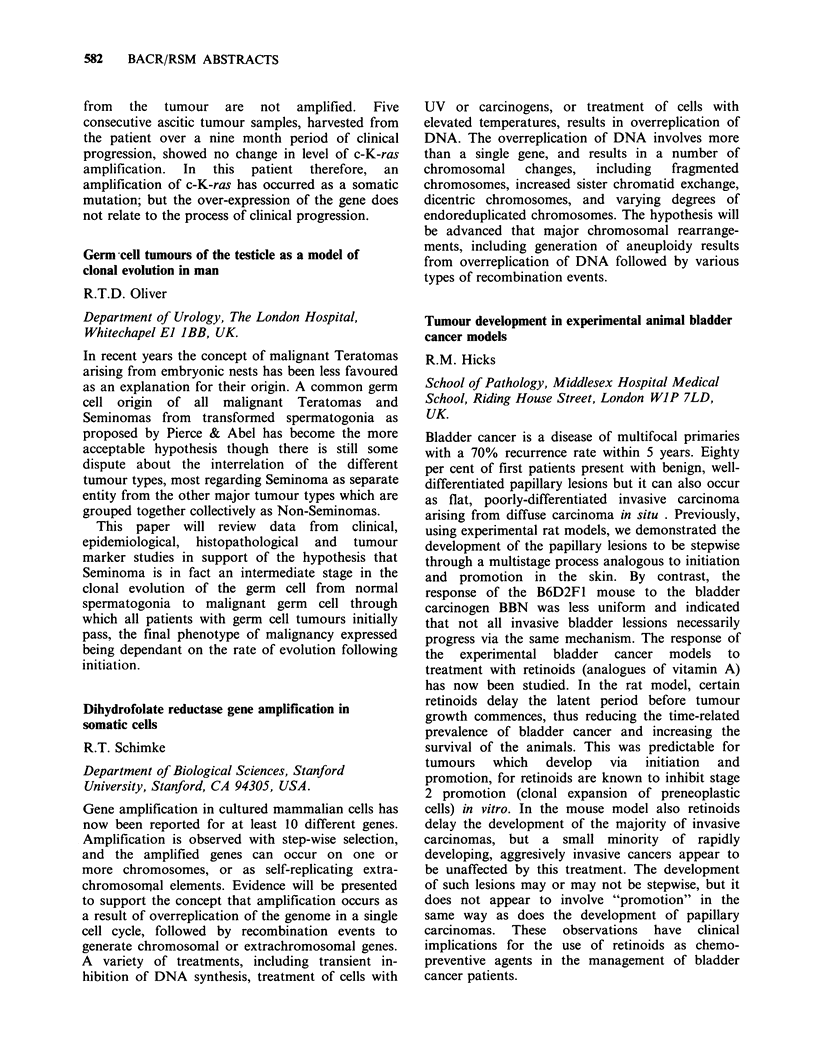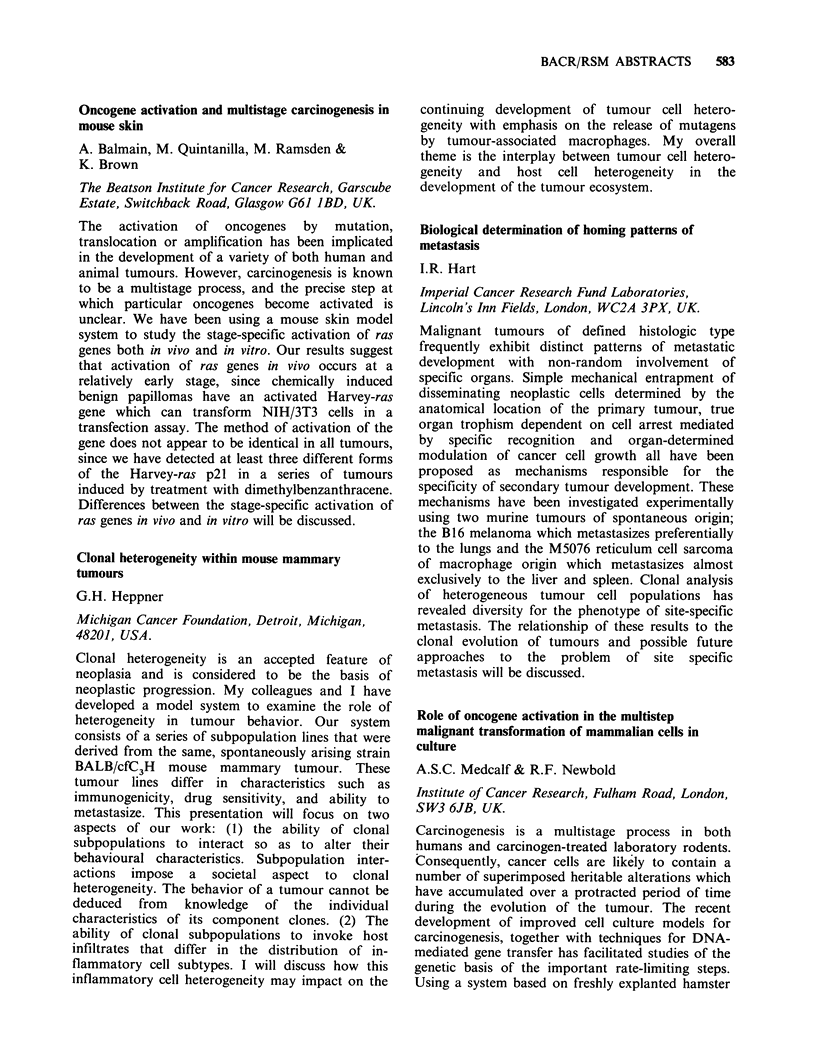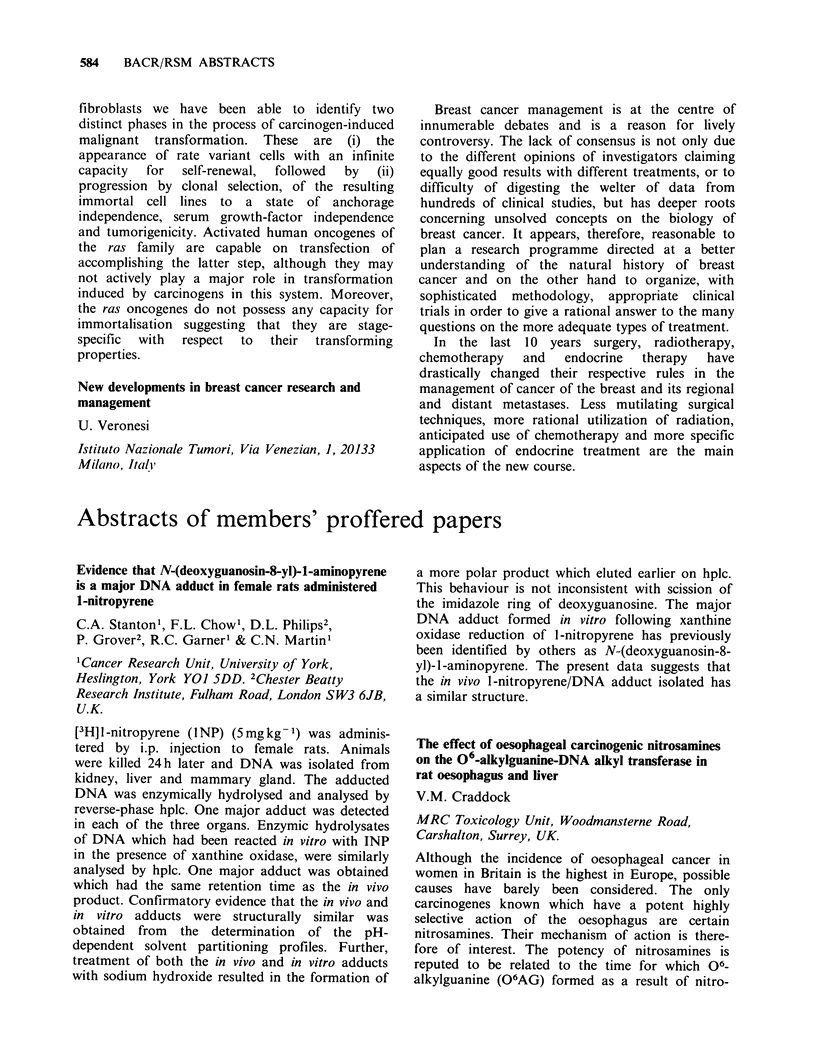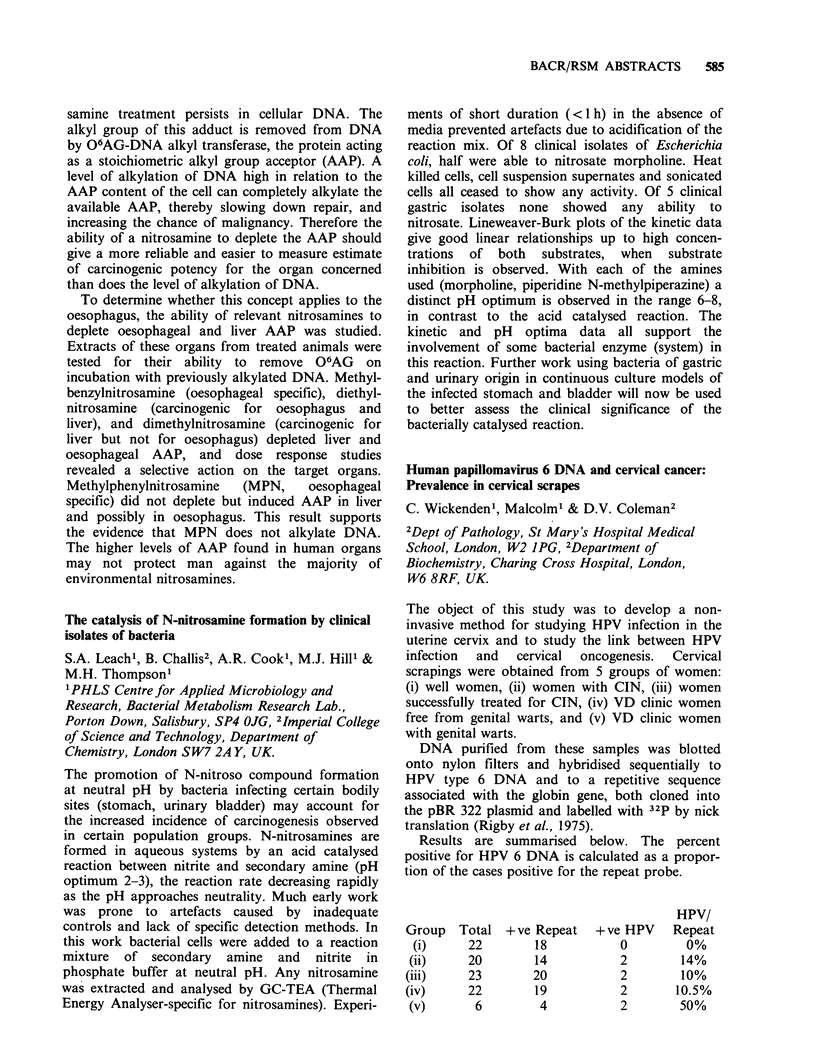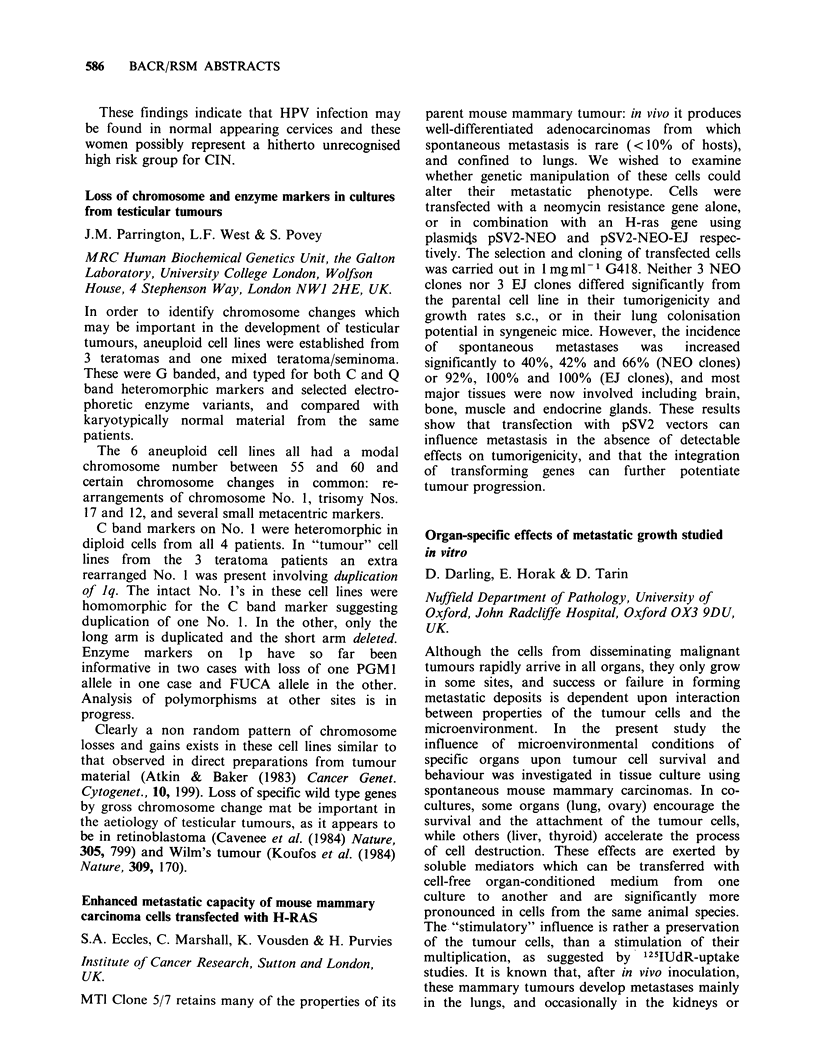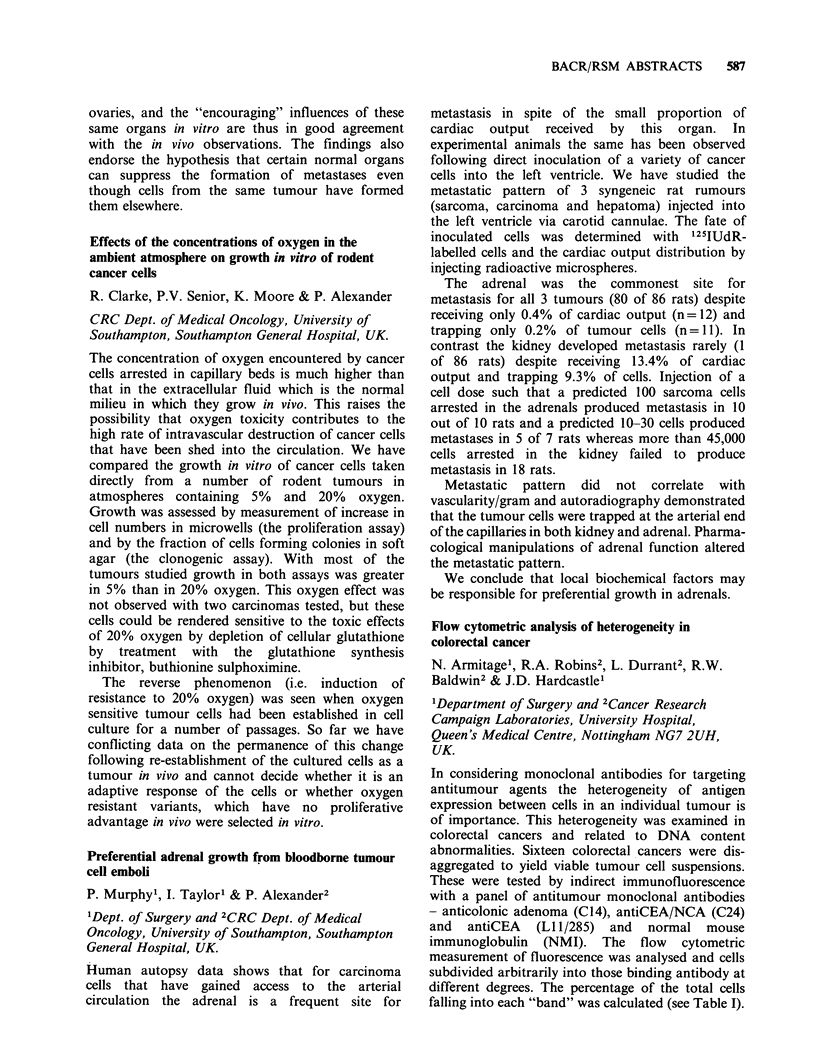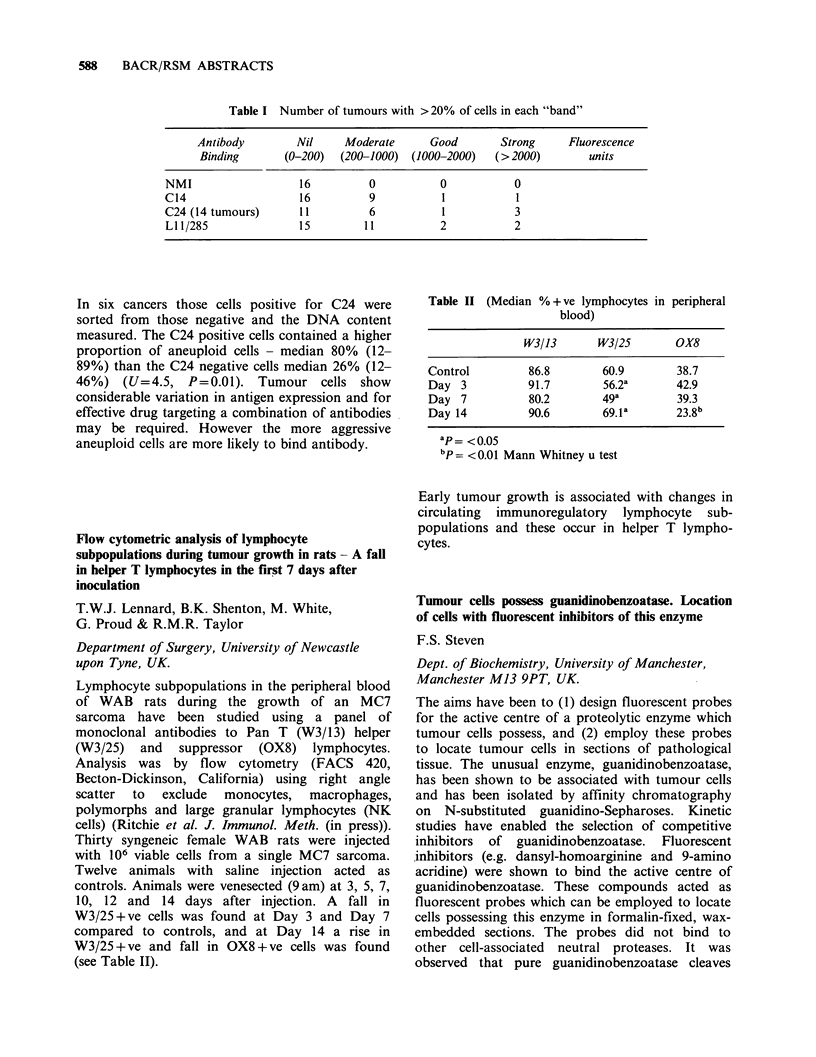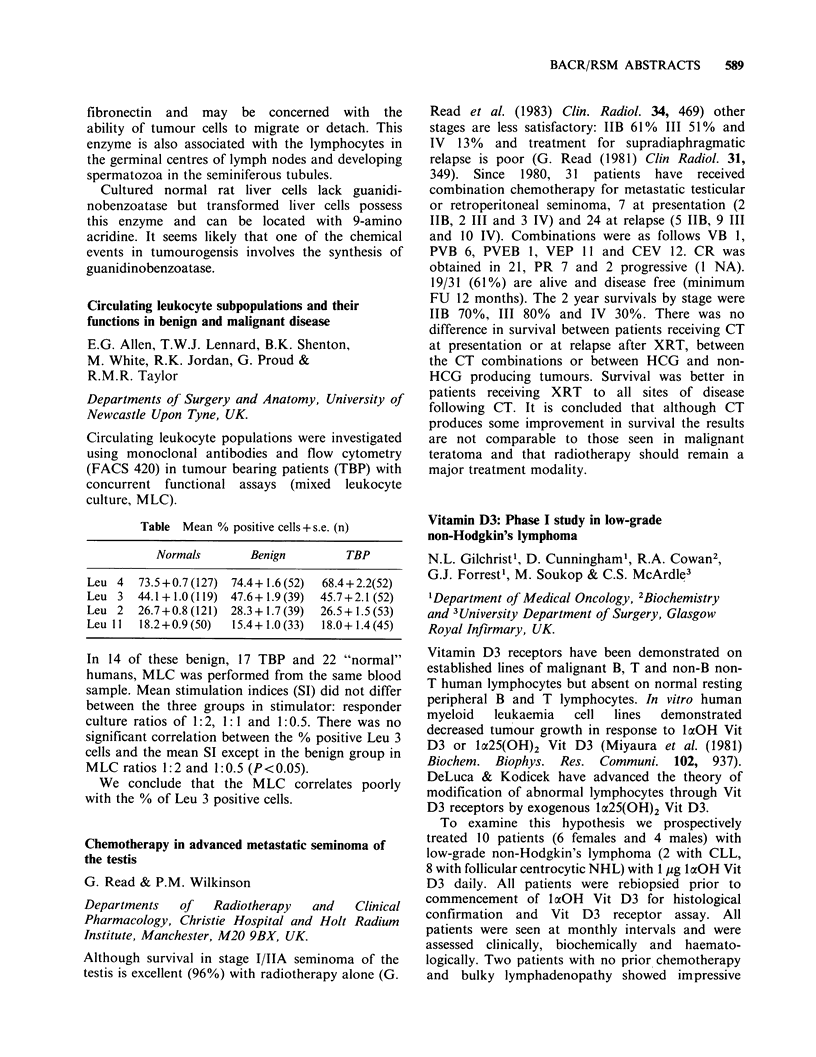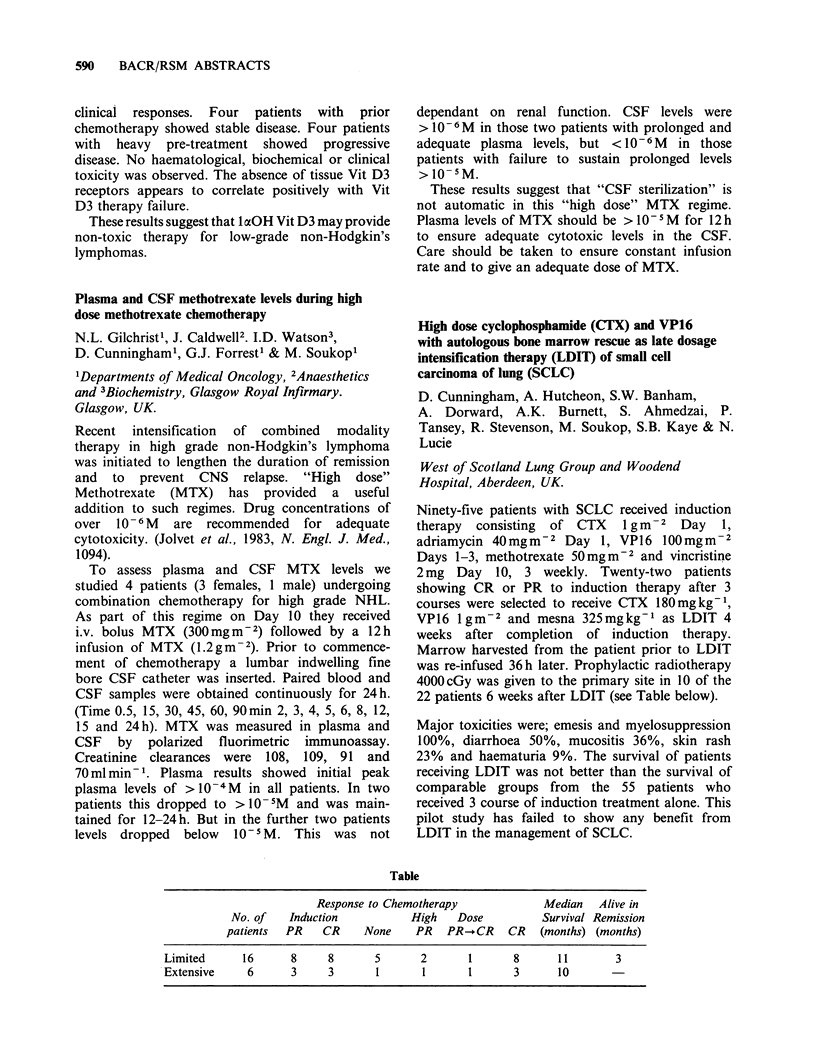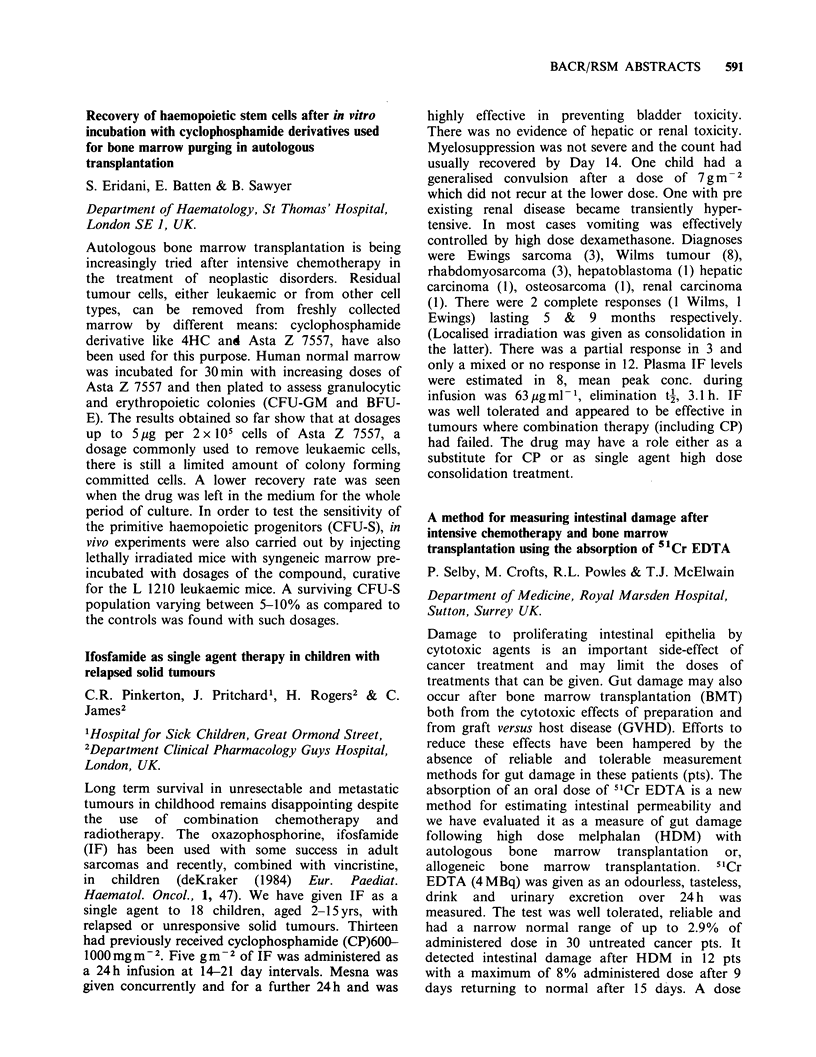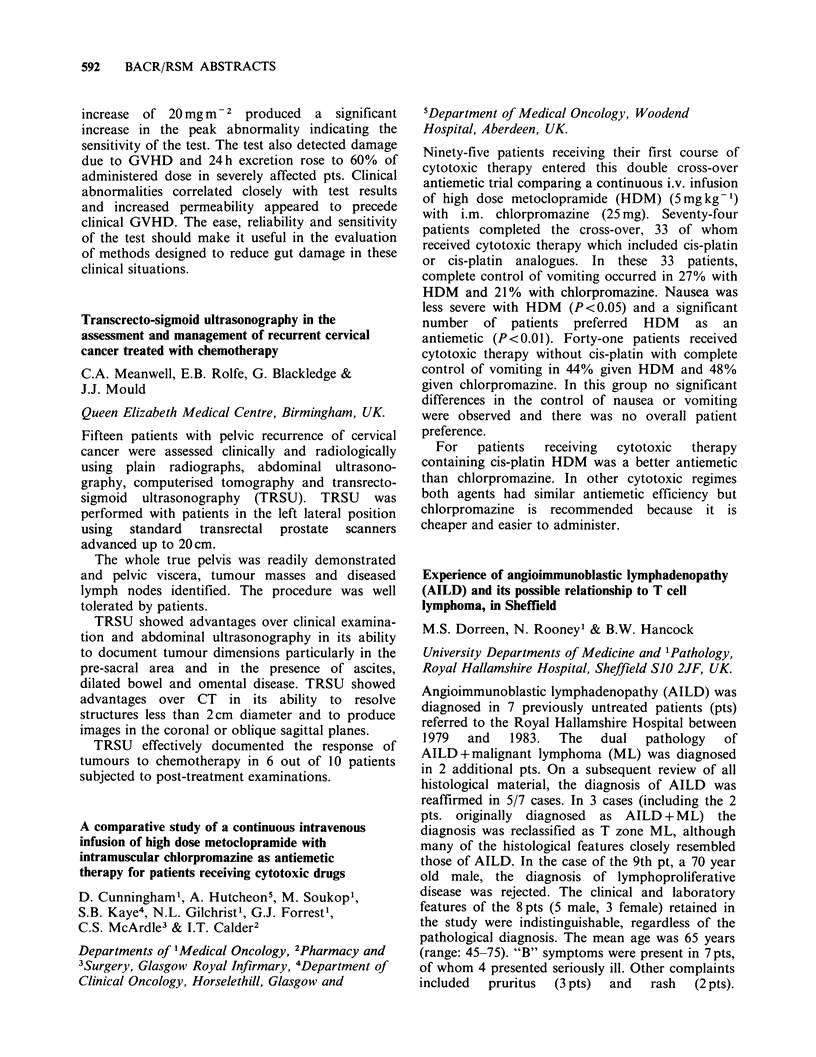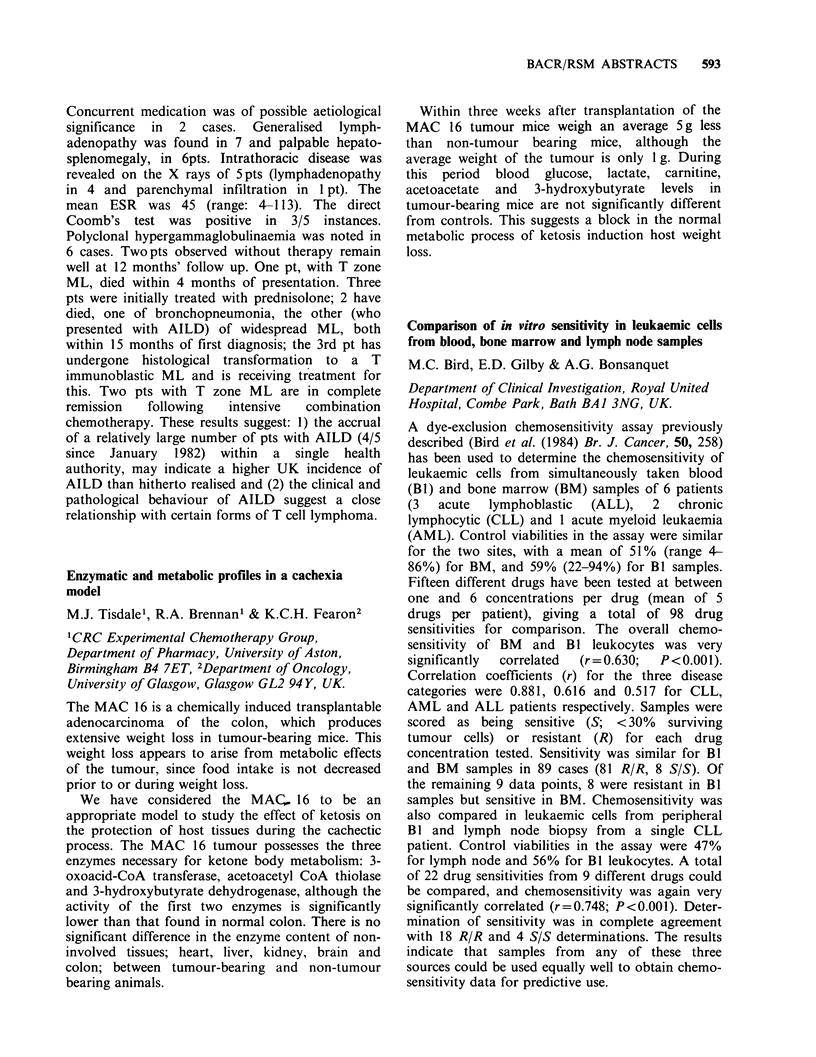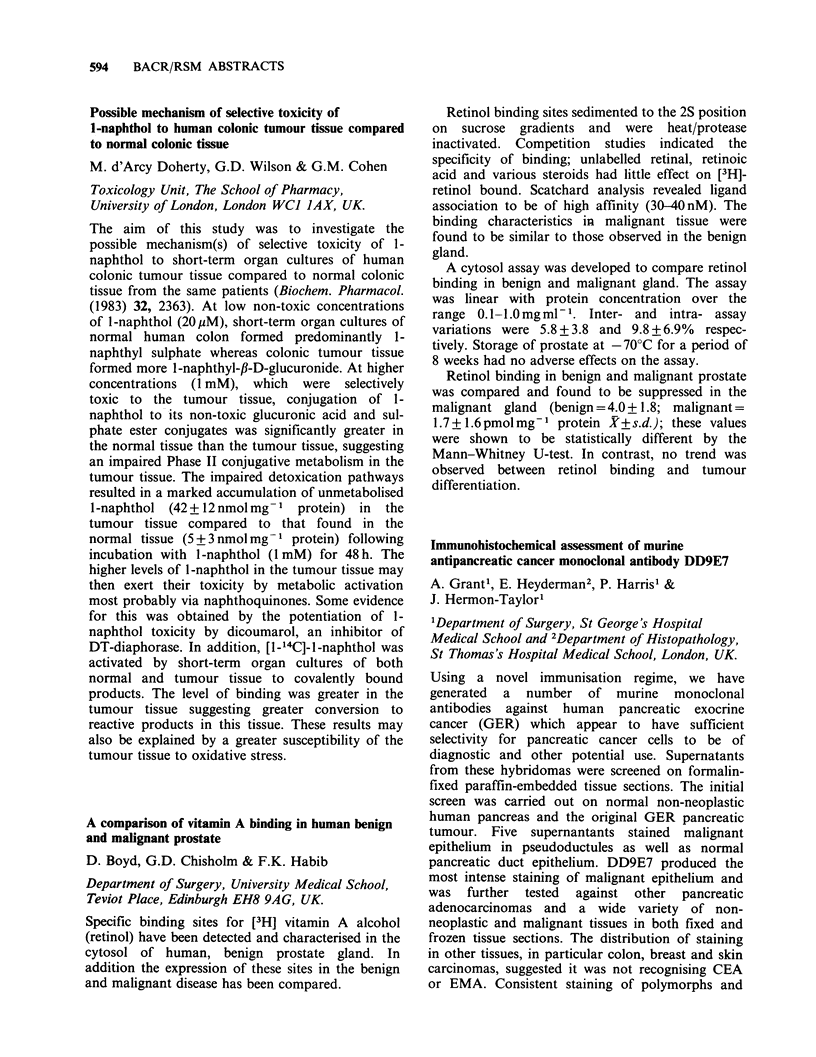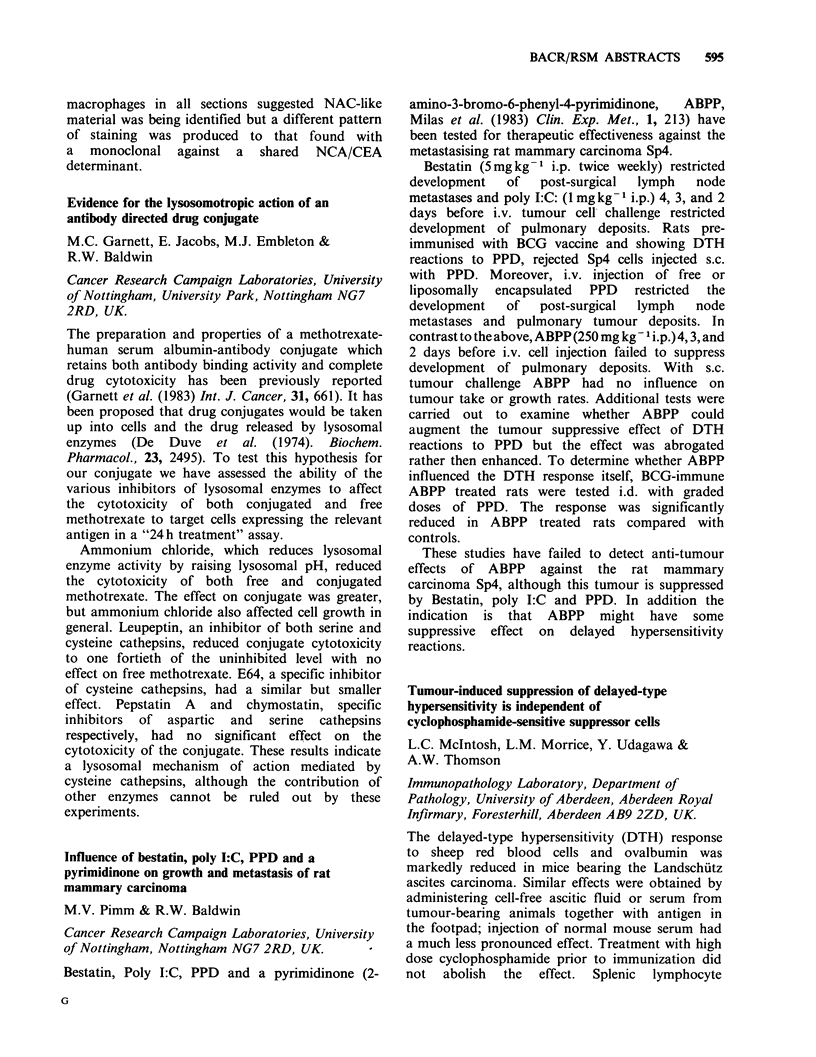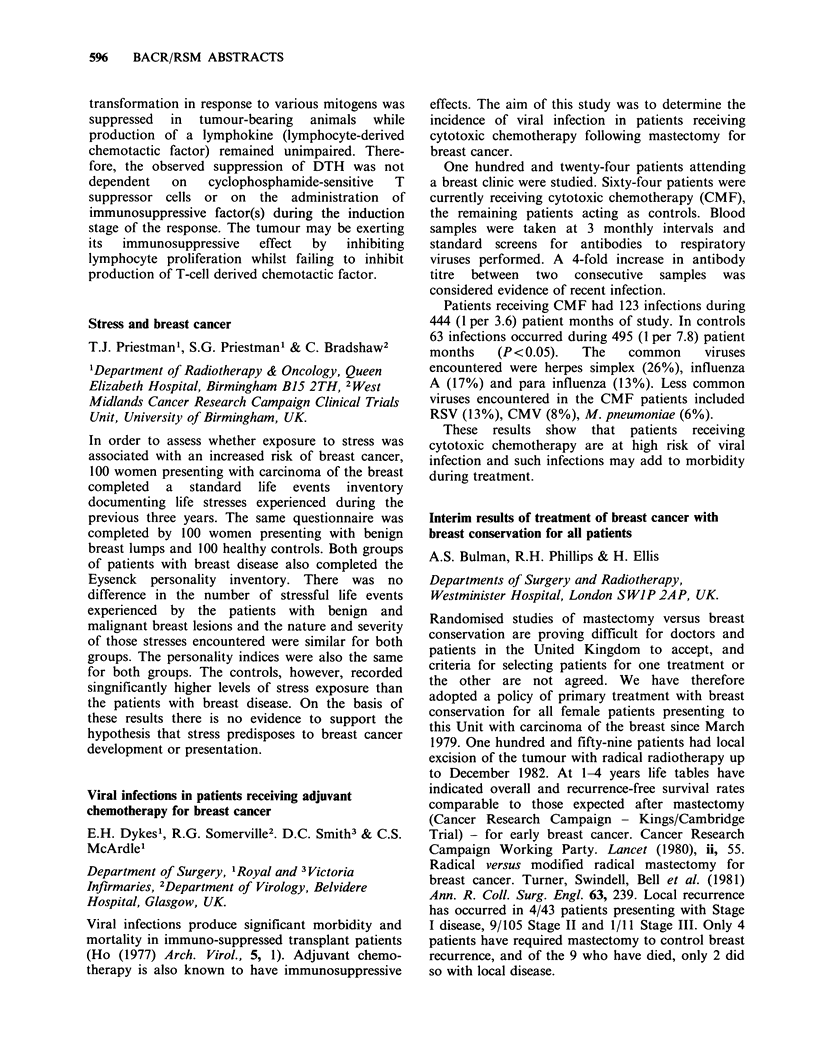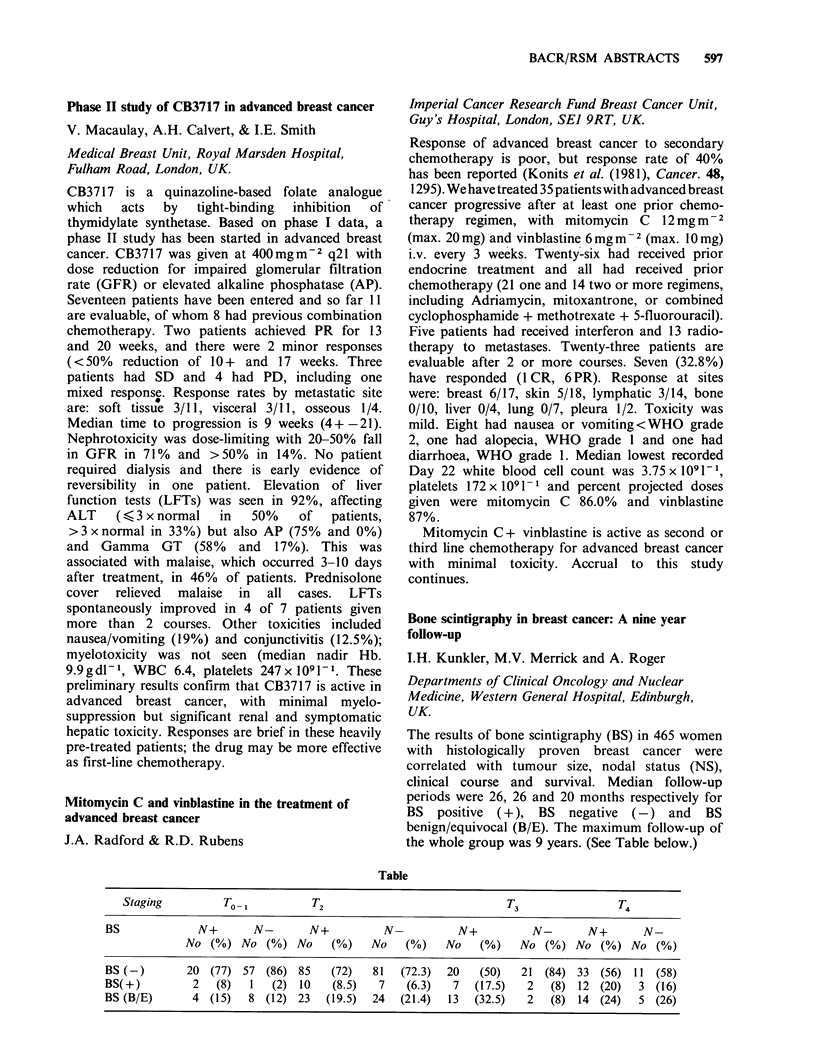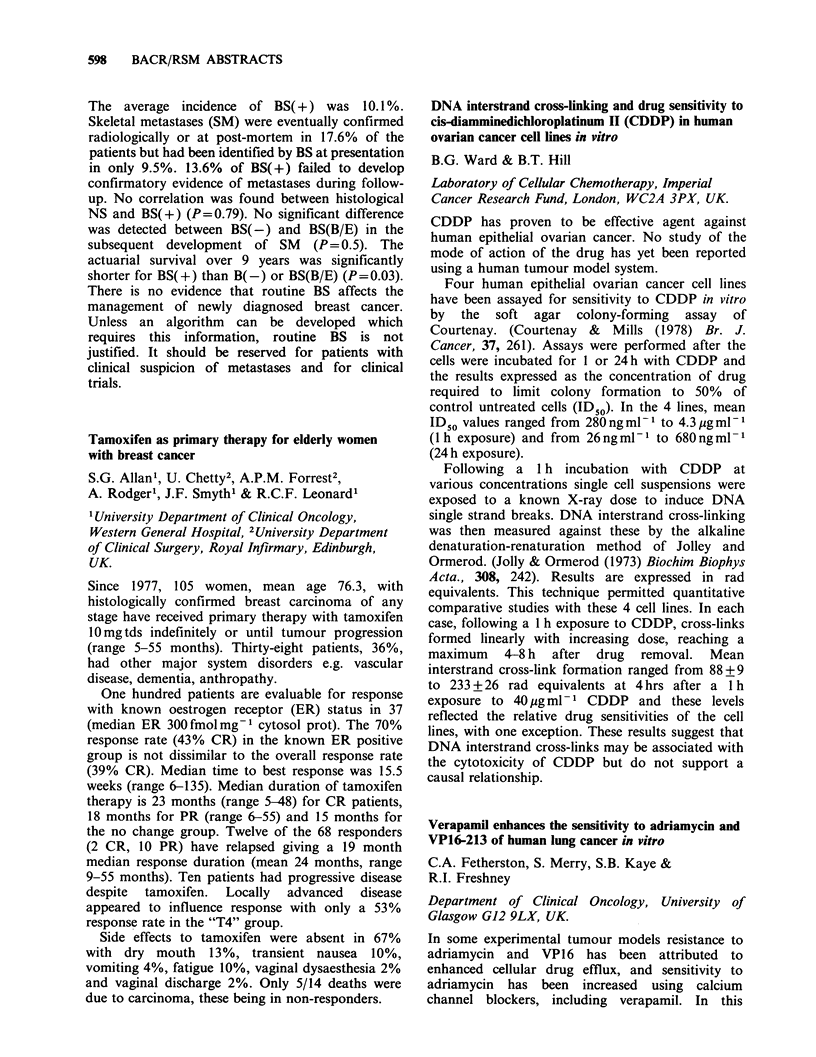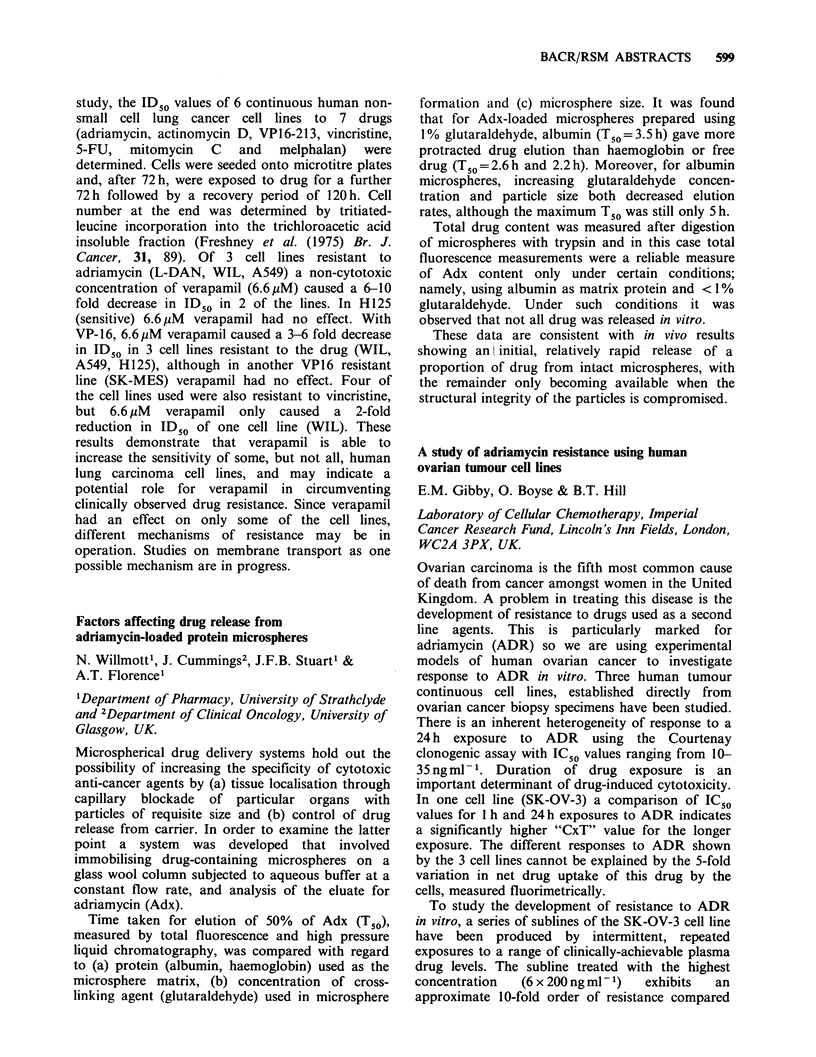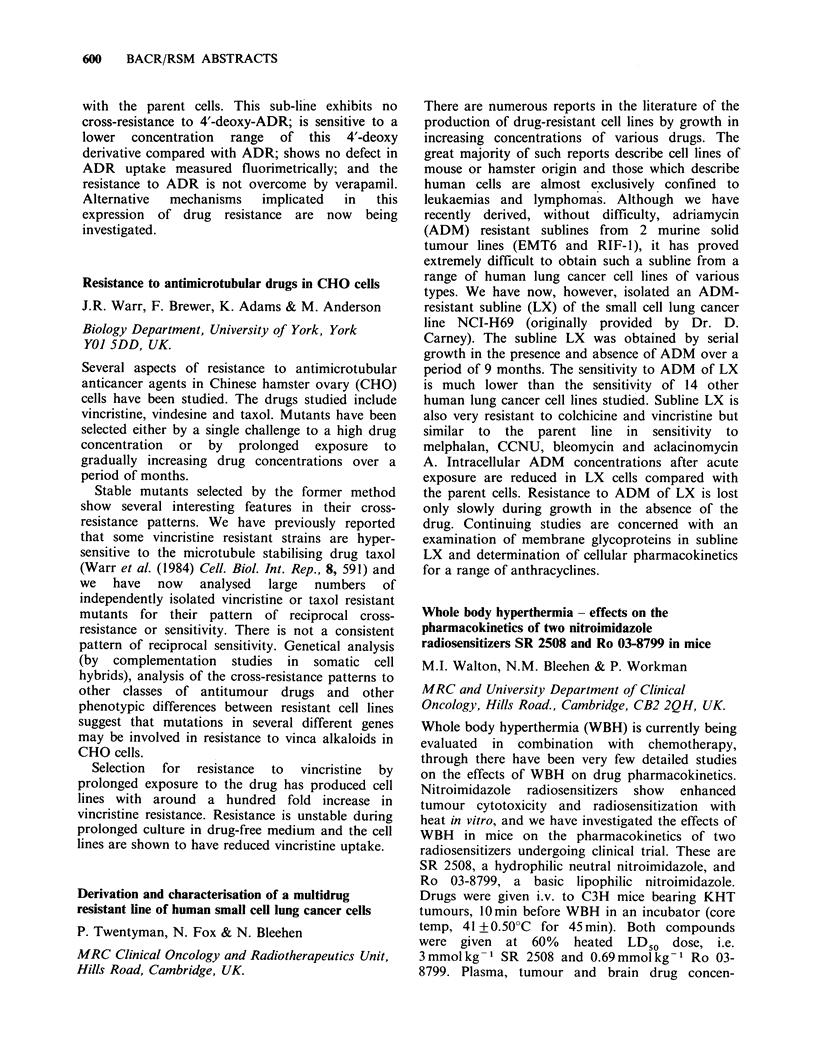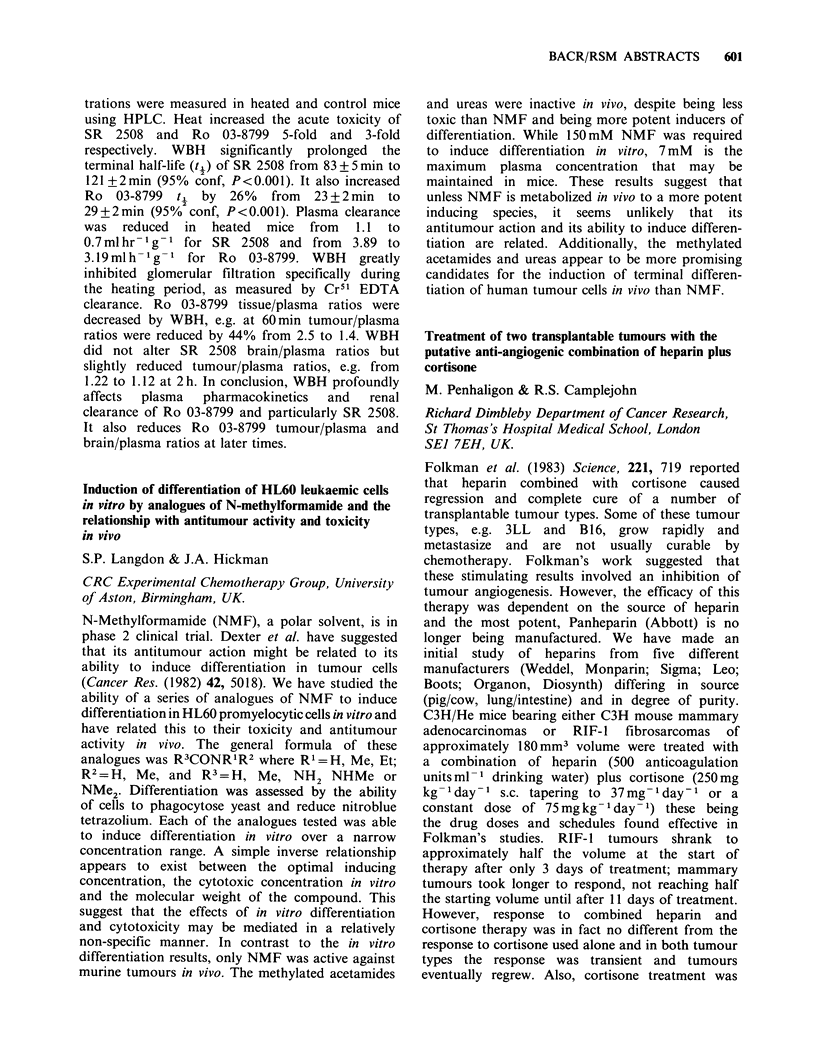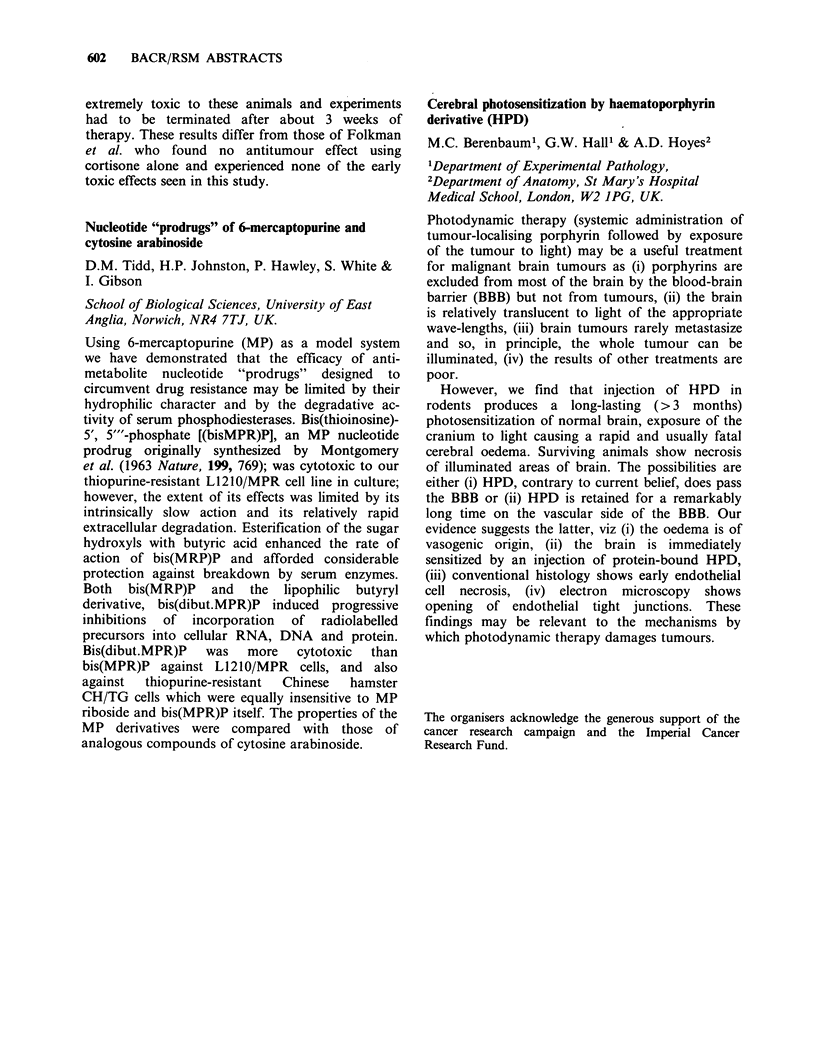# Joint meeting of the British Association for Cancer Research & the Royal Society of Medicine (Section of Oncology) (incorporating a symposium on "Clonal evolution of tumours"). November 22-23, 1984. Abstracts.

**DOI:** 10.1038/bjc.1985.82

**Published:** 1985-04

**Authors:** 


*Br. J. Cancer* (1985), **51**, 581–602

### Joint meeting of the British Association for Cancer Research* & the Royal Society of Medicine (Section of Oncology)

### (Incorporating a Symposium on "Clonal evolution of tumours", the Fifth Gordon Hamilton-Fairley Memorial Lecture‡ and the Fifth Alexander Haddow Memorial Lecture). November 22–23, 1984

*Held at the Forum Hotel, Cromwell Road, London, SW7*

### Abstracts of Invited papers†

### Tracing clonal development and cell lineages in human tumours

P.J. Fialkow

*Department of Medicine, University of Washington, Seattle, WA 98195, USA.*

Clonal development of neoplasms and hierarchal stem cell relationships can be studied conveniently in people who have two genetically distinct types of cells. Especially useful for this purpose is the cellular mosaicism in women heterozygous for the X-chromosome-linked glucose-6-phosphate dehydrogenase (G6PD). Because the G6PD locus undergoes X-chromosome inactivation, only one of the two G6PD genes is active in each somatic cell. Therefore, women heterozygous for the usual B gene ($Gd^{B}$) and a variant such as $Gd^{A}$ have two cell populations – one synthesizing B-type G6PD and the other, A-type enzyme. Tumours with a single-cell (clonal) development exhibit only one type of G6PD, but those arising from many cells may show both B and A enzymes. Only one type of G6PD was detected in granulocytes, red cells and platelets from 30 females heterozygous for G6PD who had chronic myelocytic leukaemia (CML), indicating that this disorder involves multipotent marrow stem cells and that it develops clonally. Detailed studies with G6PD indicate that B-lymphoid and perhaps T-lymphoid cells arise from the stem cell involved by the leukaemia. Acute myeloid leukaemia is heterogeneous with respect to the pattern of stem cell involvement and the nature of remission. In some patients, the disease involves stem cells with multipotent differentiative expression, whereas in others it involves progenitors with differentiative expression restricted to the granulocytic pathway. G6PD and chromosome studies also suggest that the myeloid leukaemias have a multistep pathogenesis with clonal proliferation of marrow cells preceding acquisition of distinctive chromosomal abnormalities. Evidence for clonal development has also been adduced for many but not all of the other human tumours studied with G6PD.

### Investigations of a stem cell model for human cancer; cell-renewal, differentiation and oncogene expression in human ovarian cancer

R.N. Buick

*Ontario Cancer Institute and Department of Medical Biophysics, University of Toronto, Toronto, Ontario, Canada, M4X 1K9*

We have investigated the possibility of stem cell renewal and differentiation in human malignant epithelium. This has been performed both at the theoretical level and practically by assessing cellular features of human ovarian carcinomas. Cells derived from this type of tumour are heterogeneous with respect to a number of functional and phenotypic markers (labelling index, clonogenicity in tissue culture, histochemically-marked differentiation, cell surface expression of ovarian tumour-associated antigens). The fact that physical properties of the cells also change with differentiation has allowed the fractionation of tumour cell populations (on the basis of density and/or volume) and the putative ordering of differentiation markers. We have used these techniques to study the process of tumour progression in one patient. A model has evolved of a clonal differentiation hierarchy based on analysis of individual cell proliferative potential and cell differentiation state.

During a screen of ovarian carcinoma samples for activation of protoncogenes, we have identified a single case of ovarian carcinoma in which there is an amplification ($\simeq$25-fold) and over-expression of the protoncogene c-K-*ras*. Diploid cells purified

*Enquiries to the BACR Secretariat, c/o Institute of Biology, 20 Queensberry Place, London SW7 2DZ, UK.
†Reprints of these abstracts are not available – Ed.
‡This issue, pp. 459–464.

© The Macmillan Press Ltd., 1985

from the tumour are not amplified. Five consecutive ascitic tumour samples, harvested from the patient over a nine month period of clinical progression, showed no change in level of c-K-*ras* amplification. In this patient therefore, an amplification of c-K-*ras* has occurred as a somatic mutation; but the over-expression of the gene does not relate to the process of clinical progression.

**Germ cell tumours of the testicle as a model of clonal evolution in man**

R.T.D. Oliver

*Department of Urology, The London Hospital, Whitechapel E1 1BB, UK.*

In recent years the concept of malignant Teratomas arising from embryonic nests has been less favoured as an explanation for their origin. A common germ cell origin of all malignant Teratomas and Seminomas from transformed spermatogonia as proposed by Pierce & Abel has become the more acceptable hypothesis though there is still some dispute about the interrelation of the different tumour types, most regarding Seminoma as separate entity from the other major tumour types which are grouped together collectively as Non-Seminomas.

This paper will review data from clinical, epidemiological, histopathological and tumour marker studies in support of the hypothesis that Seminoma is in fact an intermediate stage in the clonal evolution of the germ cell from normal spermatogonia to malignant germ cell through which all patients with germ cell tumours initially pass, the final phenotype of malignancy expressed being dependant on the rate of evolution following initiation.

**Dihydrofolate reductase gene amplification in somatic cells**

R.T. Schimke

*Department of Biological Sciences, Stanford University, Stanford, CA 94305, USA.*

Gene amplification in cultured mammalian cells has now been reported for at least 10 different genes. Amplification is observed with step-wise selection, and the amplified genes can occur on one or more chromosomes, or as self-replicating extra-chromosomal elements. Evidence will be presented to support the concept that amplification occurs as a result of overreplication of the genome in a single cell cycle, followed by recombination events to generate chromosomal or extrachromosomal genes. A variety of treatments, including transient inhibition of DNA synthesis, treatment of cells with UV or carcinogens, or treatment of cells with elevated temperatures, results in overreplication of DNA. The overreplication of DNA involves more than a single gene, and results in a number of chromosomal changes, including fragmented chromosomes, increased sister chromatid exchange, dicentric chromosomes, and varying degrees of endoreduplicated chromosomes. The hypothesis will be advanced that major chromosomal rearrangements, including generation of aneuploidy results from overreplication of DNA followed by various types of recombination events.

**Tumour development in experimental animal bladder cancer models**

R.M. Hicks

*School of Pathology, Middlesex Hospital Medical School, Riding House Street, London W1P 7LD, UK.*

Bladder cancer is a disease of multifocal primaries with a 70% recurrence rate within 5 years. Eighty per cent of first patients present with benign, well-differentiated papillary lesions but it can also occur as flat, poorly-differentiated invasive carcinoma arising from diffuse carcinoma *in situ* . Previously, using experimental rat models, we demonstrated the development of the papillary lesions to be stepwise through a multistage process analogous to initiation and promotion in the skin. By contrast, the response of the B6D2F1 mouse to the bladder carcinogen BBN was less uniform and indicated that not all invasive bladder lessions necessarily progress via the same mechanism. The response of the experimental bladder cancer models to treatment with retinoids (analogues of vitamin A) has now been studied. In the rat model, certain retinoids delay the latent period before tumour growth commences, thus reducing the time-related prevalence of bladder cancer and increasing the survival of the animals. This was predictable for tumours which develop via initiation and promotion, for retinoids are known to inhibit stage 2 promotion (clonal expansion of preneoplastic cells) *in vitro*. In the mouse model also retinoids delay the development of the majority of invasive carcinomas, but a small minority of rapidly developing, aggresively invasive cancers appear to be unaffected by this treatment. The development of such lesions may or may not be stepwise, but it does not appear to involve "promotion" in the same way as does the development of papillary carcinomas. These observations have clinical implications for the use of retinoids as chemopreventive agents in the management of bladder cancer patients.

### Oncogene activation and multistage carcinogenesis in mouse skin

A. Balmain, M. Quintanilla, M. Ramsden & K. Brown

*The Beatson Institute for Cancer Research, Garscube Estate, Switchback Road, Glasgow G61 1BD, UK.*

The activation of oncogenes by mutation, translocation or amplification has been implicated in the development of a variety of both human and animal tumours. However, carcinogenesis is known to be a multistage process, and the precise step at which particular oncogenes become activated is unclear. We have been using a mouse skin model system to study the stage-specific activation of *ras* genes both *in vivo* and *in vitro*. Our results suggest that activation of *ras* genes *in vivo* occurs at a relatively early stage, since chemically induced benign papillomas have an activated Harvey-*ras* gene which can transform NIH/3T3 cells in a transfection assay. The method of activation of the gene does not appear to be identical in all tumours, since we have detected at least three different forms of the Harvey-*ras* p21 in a series of tumours induced by treatment with dimethylbenzanthracene. Differences between the stage-specific activation of *ras* genes *in vivo* and *in vitro* will be discussed.

### Clonal heterogeneity within mouse mammary tumours

G.H. Heppner

*Michigan Cancer Foundation, Detroit, Michigan, 48201, USA.*

Clonal heterogeneity is an accepted feature of neoplasia and is considered to be the basis of neoplastic progression. My colleagues and I have developed a model system to examine the role of heterogeneity in tumour behavior. Our system consists of a series of subpopulation lines that were derived from the same, spontaneously arising strain BALB/cfC$_3$H mouse mammary tumour. These tumour lines differ in characteristics such as immunogenicity, drug sensitivity, and ability to metastasize. This presentation will focus on two aspects of our work: (1) the ability of clonal subpopulations to interact so as to alter their behavioural characteristics. Subpopulation interactions impose a societal aspect to clonal heterogeneity. The behavior of a tumour cannot be deduced from knowledge of the individual characteristics of its component clones. (2) The ability of clonal subpopulations to invoke host infiltrates that differ in the distribution of inflammatory cell subtypes. I will discuss how this inflammatory cell heterogeneity may impact on the continuing development of tumour cell heterogeneity with emphasis on the release of mutagens by tumour-associated macrophages. My overall theme is the interplay between tumour cell heterogeneity and host cell heterogeneity in the development of the tumour ecosystem.

### Biological determination of homing patterns of metastasis

I.R. Hart

*Imperial Cancer Research Fund Laboratories, Lincoln's Inn Fields, London, WC2A 3PX, UK.*

Malignant tumours of defined histologic type frequently exhibit distinct patterns of metastatic development with non-random involvement of specific organs. Simple mechanical entrapment of disseminating neoplastic cells determined by the anatomical location of the primary tumour, true organ trophism dependent on cell arrest mediated by specific recognition and organ-determined modulation of cancer cell growth all have been proposed as mechanisms responsible for the specificity of secondary tumour development. These mechanisms have been investigated experimentally using two murine tumours of spontaneous origin; the B16 melanoma which metastasizes preferentially to the lungs and the M5076 reticulum cell sarcoma of macrophage origin which metastasizes almost exclusively to the liver and spleen. Clonal analysis of heterogeneous tumour cell populations has revealed diversity for the phenotype of site-specific metastasis. The relationship of these results to the clonal evolution of tumours and possible future approaches to the problem of site specific metastasis will be discussed.

### Role of oncogene activation in the multistep malignant transformation of mammalian cells in culture

A.S.C. Medcalf & R.F. Newbold

*Institute of Cancer Research, Fulham Road, London, SW3 6JB, UK.*

Carcinogenesis is a multistage process in both humans and carcinogen-treated laboratory rodents. Consequently, cancer cells are likely to contain a number of superimposed heritable alterations which have accumulated over a protracted period of time during the evolution of the tumour. The recent development of improved cell culture models for carcinogenesis, together with techniques for DNA-mediated gene transfer has facilitated studies of the genetic basis of the important rate-limiting steps. Using a system based on freshly explanted hamster fibroblasts we have been able to identify two distinct phases in the process of carcinogen-induced malignant transformation. These are (i) the appearance of rate variant cells with an infinite capacity for self-renewal, followed by (ii) progression by clonal selection, of the resulting immortal cell lines to a state of anchorage independence, serum growth-factor independence and tumorigenicity. Activated human oncogenes of the *ras* family are capable on transfection of accomplishing the latter step, although they may not actively play a major role in transformation induced by carcinogens in this system. Moreover, the *ras* oncogenes do not possess any capacity for immortalisation suggesting that they are stage-specific with respect to their transforming properties.

**New developments in breast cancer research and management**

U. Veronesi

*Istituto Nazionale Tumori, Via Venezian, 1, 20133 Milano, Italy*

Breast cancer management is at the centre of innumerable debates and is a reason for lively controversy. The lack of consensus is not only due to the different opinions of investigators claiming equally good results with different treatments, or to difficulty of digesting the welter of data from hundreds of clinical studies, but has deeper roots concerning unsolved concepts on the biology of breast cancer. It appears, therefore, reasonable to plan a research programme directed at a better understanding of the natural history of breast cancer and on the other hand to organize, with sophisticated methodology, appropriate clinical trials in order to give a rational answer to the many questions on the more adequate types of treatment.

In the last 10 years surgery, radiotherapy, chemotherapy and endocrine therapy have drastically changed their respective rules in the management of cancer of the breast and its regional and distant metastases. Less mutilating surgical techniques, more rational utilization of radiation, anticipated use of chemotherapy and more specific application of endocrine treatment are the main aspects of the new course.

### Abstracts of members' proffered papers

**Evidence that *N*-(deoxyguanosin-8-yl)-1-aminopyrene is a major DNA adduct in female rats administered 1-nitropyrene**

C.A. Stanton[1], F.L. Chow[1], D.L. Philips[2], P. Grover[2], R.C. Garner[1] & C.N. Martin[1]

[1]*Cancer Research Unit, University of York, Heslington, York YO1 5DD.* [2]*Chester Beatty Research Institute, Fulham Road, London SW3 6JB, U.K.*

[$^3$H]1-nitropyrene (1NP) (5 mg kg$^{-1}$) was administered by i.p. injection to female rats. Animals were killed 24 h later and DNA was isolated from kidney, liver and mammary gland. The adducted DNA was enzymically hydrolysed and analysed by reverse-phase hplc. One major adduct was detected in each of the three organs. Enzymic hydrolysates of DNA which had been reacted *in vitro* with INP in the presence of xanthine oxidase, were similarly analysed by hplc. One major adduct was obtained which had the same retention time as the *in vivo* product. Confirmatory evidence that the *in vivo* and *in vitro* adducts were structurally similar was obtained from the determination of the pH-dependent solvent partitioning profiles. Further, treatment of both the *in vivo* and *in vitro* adducts with sodium hydroxide resulted in the formation of a more polar product which eluted earlier on hplc. This behaviour is not inconsistent with scission of the imidazole ring of deoxyguanosine. The major DNA adduct formed *in vitro* following xanthine oxidase reduction of 1-nitropyrene has previously been identified by others as *N*-(deoxyguanosin-8-yl)-1-aminopyrene. The present data suggests that the *in vivo* 1-nitropyrene/DNA adduct isolated has a similar structure.

**The effect of oesophageal carcinogenic nitrosamines on the O$^6$-alkylguanine-DNA alkyl transferase in rat oesophagus and liver**

V.M. Craddock

*MRC Toxicology Unit, Woodmansterne Road, Carshalton, Surrey, UK.*

Although the incidence of oesophageal cancer in women in Britain is the highest in Europe, possible causes have barely been considered. The only carcinogenes known which have a potent highly selective action of the oesophagus are certain nitrosamines. Their mechanism of action is therefore of interest. The potency of nitrosamines is reputed to be related to the time for which O$^6$-alkylguanine (O$^6$AG) formed as a result of nitrosamine treatment persists in cellular DNA. The alkyl group of this adduct is removed from DNA by O[6]AG-DNA alkyl transferase, the protein acting as a stoichiometric alkyl group acceptor (AAP). A level of alkylation of DNA high in relation to the AAP content of the cell can completely alkylate the available AAP, thereby slowing down repair, and increasing the chance of malignancy. Therefore the ability of a nitrosamine to deplete the AAP should give a more reliable and easier to measure estimate of carcinogenic potency for the organ concerned than does the level of alkylation of DNA.

To determine whether this concept applies to the oesophagus, the ability of relevant nitrosamines to deplete oesophageal and liver AAP was studied. Extracts of these organs from treated animals were tested for their ability to remove O[6]AG on incubation with previously alkylated DNA. Methylbenzylnitrosamine (oesophageal specific), diethylnitrosamine (carcinogenic for oesophagus and liver), and dimethylnitrosamine (carcinogenic for liver but not for oesophagus) depleted liver and oesophageal AAP, and dose response studies revealed a selective action on the target organs. Methylphenylnitrosamine (MPN, oesophageal specific) did not deplete but induced AAP in liver and possibly in oesophagus. This result supports the evidence that MPN does not alkylate DNA. The higher levels of AAP found in human organs may not protect man against the majority of environmental nitrosamines.

### The catalysis of N-nitrosamine formation by clinical isolates of bacteria

S.A. Leach[1], B. Challis[2], A.R. Cook[1], M.J. Hill[1] & M.H. Thompson[1]

[1]*PHLS Centre for Applied Microbiology and Research, Bacterial Metabolism Research Lab., Porton Down, Salisbury, SP4 0JG,* [2]*Imperial College of Science and Technology, Department of Chemistry, London SW7 2AY, UK.*

The promotion of N-nitroso compound formation at neutral pH by bacteria infecting certain bodily sites (stomach, urinary bladder) may account for the increased incidence of carcinogenesis observed in certain population groups. N-nitrosamines are formed in aqueous systems by an acid catalysed reaction between nitrite and secondary amine (pH optimum 2–3), the reaction rate decreasing rapidly as the pH approaches neutrality. Much early work was prone to artefacts caused by inadequate controls and lack of specific detection methods. In this work bacterial cells were added to a reaction mixture of secondary amine and nitrite in phosphate buffer at neutral pH. Any nitrosamine was extracted and analysed by GC-TEA (Thermal Energy Analyser-specific for nitrosamines). Experiments of short duration (<1 h) in the absence of media prevented artefacts due to acidification of the reaction mix. Of 8 clinical isolates of *Escherichia coli*, half were able to nitrosate morpholine. Heat killed cells, cell suspension supernates and sonicated cells all ceased to show any activity. Of 5 clinical gastric isolates none showed any ability to nitrosate. Lineweaver-Burk plots of the kinetic data give good linear relationships up to high concentrations of both substrates, when substrate inhibition is observed. With each of the amines used (morpholine, piperidine N-methylpiperazine) a distinct pH optimum is observed in the range 6–8, in contrast to the acid catalysed reaction. The kinetic and pH optima data all support the involvement of some bacterial enzyme (system) in this reaction. Further work using bacteria of gastric and urinary origin in continuous culture models of the infected stomach and bladder will now be used to better assess the clinical significance of the bacterially catalysed reaction.

### Human papillomavirus 6 DNA and cervical cancer: Prevalence in cervical scrapes

C. Wickenden[1], Malcolm[1] & D.V. Coleman[2]

[2]*Dept of Pathology, St Mary's Hospital Medical School, London, W2 1PG,* [2]*Department of Biochemistry, Charing Cross Hospital, London, W6 8RF, UK.*

The object of this study was to develop a non-invasive method for studying HPV infection in the uterine cervix and to study the link between HPV infection and cervical oncogenesis. Cervical scrapings were obtained from 5 groups of women: (i) well women, (ii) women with CIN, (iii) women successfully treated for CIN, (iv) VD clinic women free from genital warts, and (v) VD clinic women with genital warts.

DNA purified from these samples was blotted onto nylon filters and hybridised sequentially to HPV type 6 DNA and to a repetitive sequence associated with the globin gene, both cloned into the pBR 322 plasmid and labelled with $^{32}P$ by nick translation (Rigby *et al.*, 1975).

Results are summarised below. The percent positive for HPV 6 DNA is calculated as a proportion of the cases positive for the repeat probe.

| Group | Total | +ve Repeat | +ve HPV | HPV/ Repeat |
|---|---|---|---|---|
| (i) | 22 | 18 | 0 | 0% |
| (ii) | 20 | 14 | 2 | 14% |
| (iii) | 23 | 20 | 2 | 10% |
| (iv) | 22 | 19 | 2 | 10.5% |
| (v) | 6 | 4 | 2 | 50% |

These findings indicate that HPV infection may be found in normal appearing cervices and these women possibly represent a hitherto unrecognised high risk group for CIN.

### Loss of chromosome and enzyme markers in cultures from testicular tumours

J.M. Parrington, L.F. West & S. Povey

*MRC Human Biochemical Genetics Unit, the Galton Laboratory, University College London, Wolfson House, 4 Stephenson Way, London NW1 2HE, UK.*

In order to identify chromosome changes which may be important in the development of testicular tumours, aneuploid cell lines were established from 3 teratomas and one mixed teratoma/seminoma. These were G banded, and typed for both C and Q band heteromorphic markers and selected electrophoretic enzyme variants, and compared with karyotypically normal material from the same patients.

The 6 aneuploid cell lines all had a modal chromosome number between 55 and 60 and certain chromosome changes in common: rearrangements of chromosome No. 1, trisomy Nos. 17 and 12, and several small metacentric markers.

C band markers on No. 1 were heteromorphic in diploid cells from all 4 patients. In "tumour" cell lines from the 3 teratoma patients an extra rearranged No. 1 was present involving *duplication of 1q*. The intact No. 1's in these cell lines were homomorphic for the C band marker suggesting duplication of one No. 1. In the other, only the long arm is duplicated and the short arm *deleted*. Enzyme markers on 1p have so far been informative in two cases with loss of one PGM1 allele in one case and FUCA allele in the other. Analysis of polymorphisms at other sites is in progress.

Clearly a non random pattern of chromosome losses and gains exists in these cell lines similar to that observed in direct preparations from tumour material (Atkin & Baker (1983) *Cancer Genet. Cytogenet.*, **10**, 199). Loss of specific wild type genes by gross chromosome change mat be important in the aetiology of testicular tumours, as it appears to be in retinoblastoma (Cavenee *et al.* (1984) *Nature*, **305**, 799) and Wilm's tumour (Koufos *et al.* (1984) *Nature*, **309**, 170).

### Enhanced metastatic capacity of mouse mammary carcinoma cells transfected with H-RAS

S.A. Eccles, C. Marshall, K. Vousden & H. Purvies

*Institute of Cancer Research, Sutton and London, UK.*

MTl Clone 5/7 retains many of the properties of its parent mouse mammary tumour: *in vivo* it produces well-differentiated adenocarcinomas from which spontaneous metastasis is rare (<10% of hosts), and confined to lungs. We wished to examine whether genetic manipulation of these cells could alter their metastatic phenotype. Cells were transfected with a neomycin resistance gene alone, or in combination with an H-ras gene using plasmids pSV2-NEO and pSV2-NEO-EJ respectively. The selection and cloning of transfected cells was carried out in 1 mg ml$^{-1}$ G418. Neither 3 NEO clones nor 3 EJ clones differed significantly from the parental cell line in their tumorigenicity and growth rates s.c., or in their lung colonisation potential in syngeneic mice. However, the incidence of spontaneous metastases was increased significantly to 40%, 42% and 66% (NEO clones) or 92%, 100% and 100% (EJ clones), and most major tissues were now involved including brain, bone, muscle and endocrine glands. These results show that transfection with pSV2 vectors can influence metastasis in the absence of detectable effects on tumorigenicity, and that the integration of transforming genes can further potentiate tumour progression.

### Organ-specific effects of metastatic growth studied *in vitro*

D. Darling, E. Horak & D. Tarin

*Nuffield Department of Pathology, University of Oxford, John Radcliffe Hospital, Oxford OX3 9DU, UK.*

Although the cells from disseminating malignant tumours rapidly arrive in all organs, they only grow in some sites, and success or failure in forming metastatic deposits is dependent upon interaction between properties of the tumour cells and the microenvironment. In the present study the influence of microenvironmental conditions of specific organs upon tumour cell survival and behaviour was investigated in tissue culture using spontaneous mouse mammary carcinomas. In co-cultures, some organs (lung, ovary) encourage the survival and the attachment of the tumour cells, while others (liver, thyroid) accelerate the process of cell destruction. These effects are exerted by soluble mediators which can be transferred with cell-free organ-conditioned medium from one culture to another and are significantly more pronounced in cells from the same animal species. The "stimulatory" influence is rather a preservation of the tumour cells, than a stimulation of their multiplication, as suggested by $^{125}$IUdR-uptake studies. It is known that, after *in vivo* inoculation, these mammary tumours develop metastases mainly in the lungs, and occasionally in the kidneys or ovaries, and the "encouraging" influences of these same organs *in vitro* are thus in good agreement with the *in vivo* observations. The findings also endorse the hypothesis that certain normal organs can suppress the formation of metastases even though cells from the same tumour have formed them elsewhere.

### Effects of the concentrations of oxygen in the ambient atmosphere on growth *in vitro* of rodent cancer cells

R. Clarke, P.V. Senior, K. Moore & P. Alexander

*CRC Dept. of Medical Oncology, University of Southampton, Southampton General Hospital, UK.*

The concentration of oxygen encountered by cancer cells arrested in capillary beds is much higher than that in the extracellular fluid which is the normal milieu in which they grow *in vivo*. This raises the possibility that oxygen toxicity contributes to the high rate of intravascular destruction of cancer cells that have been shed into the circulation. We have compared the growth *in vitro* of cancer cells taken directly from a number of rodent tumours in atmospheres containing 5% and 20% oxygen. Growth was assessed by measurement of increase in cell numbers in microwells (the proliferation assay) and by the fraction of cells forming colonies in soft agar (the clonogenic assay). With most of the tumours studied growth in both assays was greater in 5% than in 20% oxygen. This oxygen effect was not observed with two carcinomas tested, but these cells could be rendered sensitive to the toxic effects of 20% oxygen by depletion of cellular glutathione by treatment with the glutathione synthesis inhibitor, buthionine sulphoximine.

The reverse phenomenon (i.e. induction of resistance to 20% oxygen) was seen when oxygen sensitive tumour cells had been established in cell culture for a number of passages. So far we have conflicting data on the permanence of this change following re-establishment of the cultured cells as a tumour *in vivo* and cannot decide whether it is an adaptive response of the cells or whether oxygen resistant variants, which have no proliferative advantage *in vivo* were selected *in vitro*.

### Preferential adrenal growth from bloodborne tumour cell emboli

P. Murphy[1], I. Taylor[1] & P. Alexander[2]

[1]*Dept. of Surgery and* [2]*CRC Dept. of Medical Oncology, University of Southampton, Southampton General Hospital, UK.*

Human autopsy data shows that for carcinoma cells that have gained access to the arterial circulation the adrenal is a frequent site for metastasis in spite of the small proportion of cardiac output received by this organ. In experimental animals the same has been observed following direct inoculation of a variety of cancer cells into the left ventricle. We have studied the metastatic pattern of 3 syngeneic rat rumours (sarcoma, carcinoma and hepatoma) injected into the left ventricle via carotid cannulae. The fate of inoculated cells was determined with $^{125}$IUdR-labelled cells and the cardiac output distribution by injecting radioactive microspheres.

The adrenal was the commonest site for metastasis for all 3 tumours (80 of 86 rats) despite receiving only 0.4% of cardiac output ($n=12$) and trapping only 0.2% of tumour cells ($n=11$). In contrast the kidney developed metastasis rarely (1 of 86 rats) despite receiving 13.4% of cardiac output and trapping 9.3% of cells. Injection of a cell dose such that a predicted 100 sarcoma cells arrested in the adrenals produced metastasis in 10 out of 10 rats and a predicted 10–30 cells produced metastases in 5 of 7 rats whereas more than 45,000 cells arrested in the kidney failed to produce metastasis in 18 rats.

Metastatic pattern did not correlate with vascularity/gram and autoradiography demonstrated that the tumour cells were trapped at the arterial end of the capillaries in both kidney and adrenal. Pharmacological manipulations of adrenal function altered the metastatic pattern.

We conclude that local biochemical factors may be responsible for preferential growth in adrenals.

### Flow cytometric analysis of heterogeneity in colorectal cancer

N. Armitage[1], R.A. Robins[2], L. Durrant[2], R.W. Baldwin[2] & J.D. Hardcastle[1]

[1]*Department of Surgery and* [2]*Cancer Research Campaign Laboratories, University Hospital, Queen's Medical Centre, Nottingham NG7 2UH, UK.*

In considering monoclonal antibodies for targeting antitumour agents the heterogeneity of antigen expression between cells in an individual tumour is of importance. This heterogeneity was examined in colorectal cancers and related to DNA content abnormalities. Sixteen colorectal cancers were disaggregated to yield viable tumour cell suspensions. These were tested by indirect immunofluorescence with a panel of antitumour monoclonal antibodies – anticolonic adenoma (C14), antiCEA/NCA (C24) and antiCEA (L11/285) and normal mouse immunoglobulin (NMI). The flow cytometric measurement of fluorescence was analysed and cells subdivided arbitrarily into those binding antibody at different degrees. The percentage of the total cells falling into each "band" was calculated (see Table I).

**Table I** Number of tumours with >20% of cells in each "band"

| *Antibody Binding* | *Nil (0–200)* | *Moderate (200–1000)* | *Good (1000–2000)* | *Strong (>2000)* | *Fluorescence units* |
|---|---|---|---|---|---|
| NMI | 16 | 0 | 0 | 0 | |
| C14 | 16 | 9 | 1 | 1 | |
| C24 (14 tumours) | 11 | 6 | 1 | 3 | |
| L11/285 | 15 | 11 | 2 | 2 | |

In six cancers those cells positive for C24 were sorted from those negative and the DNA content measured. The C24 positive cells contained a higher proportion of aneuploid cells – median 80% (12–89%) than the C24 negative cells median 26% (12–46%) ($U$=4.5, $P$=0.01). Tumour cells show considerable variation in antigen expression and for effective drug targeting a combination of antibodies may be required. However the more aggressive aneuploid cells are more likely to bind antibody.

### Flow cytometric analysis of lymphocyte subpopulations during tumour growth in rats – A fall in helper T lymphocytes in the first 7 days after inoculation

T.W.J. Lennard, B.K. Shenton, M. White, G. Proud & R.M.R. Taylor

*Department of Surgery, University of Newcastle upon Tyne, UK.*

Lymphocyte subpopulations in the peripheral blood of WAB rats during the growth of an MC7 sarcoma have been studied using a panel of monoclonal antibodies to Pan T (W3/13) helper (W3/25) and suppressor (OX8) lymphocytes. Analysis was by flow cytometry (FACS 420, Becton-Dickinson, California) using right angle scatter to exclude monocytes, macrophages, polymorphs and large granular lymphocytes (NK cells) (Ritchie *et al. J. Immunol. Meth.* (in press)). Thirty syngeneic female WAB rats were injected with $10^6$ viable cells from a single MC7 sarcoma. Twelve animals with saline injection acted as controls. Animals were venesected (9 am) at 3, 5, 7, 10, 12 and 14 days after injection. A fall in W3/25+ve cells was found at Day 3 and Day 7 compared to controls, and at Day 14 a rise in W3/25+ve and fall in OX8+ve cells was found (see Table II).

**Table II** (Median %+ve lymphocytes in peripheral blood)

| | *W3/13* | *W3/25* | *OX8* |
|---|---|---|---|
| Control | 86.8 | 60.9 | 38.7 |
| Day 3 | 91.7 | 56.2[a] | 42.9 |
| Day 7 | 80.2 | 49[a] | 39.3 |
| Day 14 | 90.6 | 69.1[a] | 23.8[b] |

[a]$P$= <0.05
[b]$P$= <0.01 Mann Whitney u test

Early tumour growth is associated with changes in circulating immunoregulatory lymphocyte subpopulations and these occur in helper T lymphocytes.

### Tumour cells possess guanidinobenzoatase. Location of cells with fluorescent inhibitors of this enzyme

F.S. Steven

*Dept. of Biochemistry, University of Manchester, Manchester M13 9PT, UK.*

The aims have been to (1) design fluorescent probes for the active centre of a proteolytic enzyme which tumour cells possess, and (2) employ these probes to locate tumour cells in sections of pathological tissue. The unusual enzyme, guanidinobenzoatase, has been shown to be associated with tumour cells and has been isolated by affinity chromatography on N-substituted guanidino-Sepharoses. Kinetic studies have enabled the selection of competitive inhibitors of guanidinobenzoatase. Fluorescent inhibitors (e.g. dansyl-homoarginine and 9-amino acridine) were shown to bind the active centre of guanidinobenzoatase. These compounds acted as fluorescent probes which can be employed to locate cells possessing this enzyme in formalin-fixed, wax-embedded sections. The probes did not bind to other cell-associated neutral proteases. It was observed that pure guanidinobenzoatase cleaves fibronectin and may be concerned with the ability of tumour cells to migrate or detach. This enzyme is also associated with the lymphocytes in the germinal centres of lymph nodes and developing spermatozoa in the seminiferous tubules.

Cultured normal rat liver cells lack guanidinobenzoatase but transformed liver cells possess this enzyme and can be located with 9-amino acridine. It seems likely that one of the chemical events in tumourogensis involves the synthesis of guanidinobenzoatase.

### Circulating leukocyte subpopulations and their functions in benign and malignant disease

E.G. Allen, T.W.J. Lennard, B.K. Shenton, M. White, R.K. Jordan, G. Proud & R.M.R. Taylor

*Departments of Surgery and Anatomy, University of Newcastle Upon Tyne, UK.*

Circulating leukocyte populations were investigated using monoclonal antibodies and flow cytometry (FACS 420) in tumour bearing patients (TBP) with concurrent functional assays (mixed leukocyte culture, MLC).

**Table** Mean % positive cells + s.e. (n)

| | *Normals* | *Benign* | *TBP* |
|---|---|---|---|
| Leu 4 | 73.5+0.7 (127) | 74.4+1.6 (52) | 68.4+2.2(52) |
| Leu 3 | 44.1+1.0 (119) | 47.6+1.9 (39) | 45.7+2.1 (52) |
| Leu 2 | 26.7+0.8 (121) | 28.3+1.7 (39) | 26.5+1.5 (53) |
| Leu 11 | 18.2+0.9 (50) | 15.4+1.0 (33) | 18.0+1.4 (45) |

In 14 of these benign, 17 TBP and 22 "normal" humans, MLC was performed from the same blood sample. Mean stimulation indices (SI) did not differ between the three groups in stimulator: responder culture ratios of 1:2, 1:1 and 1:0.5. There was no significant correlation between the % positive Leu 3 cells and the mean SI except in the benign group in MLC ratios 1:2 and 1:0.5 ($P<0.05$).

We conclude that the MLC correlates poorly with the % of Leu 3 positive cells.

### Chemotherapy in advanced metastatic seminoma of the testis

G. Read & P.M. Wilkinson

*Departments of Radiotherapy and Clinical Pharmacology, Christie Hospital and Holt Radium Institute, Manchester, M20 9BX, UK.*

Although survival in stage I/IIA seminoma of the testis is excellent (96%) with radiotherapy alone (G. Read *et al.* (1983) *Clin. Radiol.* **34,** 469) other stages are less satisfactory: IIB 61% III 51% and IV 13% and treatment for supradiaphragmatic relapse is poor (G. Read (1981) *Clin Radiol.* **31,** 349). Since 1980, 31 patients have received combination chemotherapy for metastatic testicular or retroperitoneal seminoma, 7 at presentation (2 IIB, 2 III and 3 IV) and 24 at relapse (5 IIB, 9 III and 10 IV). Combinations were as follows VB 1, PVB 6, PVEB 1, VEP 11 and CEV 12. CR was obtained in 21, PR 7 and 2 progressive (1 NA). 19/31 (61%) are alive and disease free (minimum FU 12 months). The 2 year survivals by stage were IIB 70%, III 80% and IV 30%. There was no difference in survival between patients receiving CT at presentation or at relapse after XRT, between the CT combinations or between HCG and non-HCG producing tumours. Survival was better in patients receiving XRT to all sites of disease following CT. It is concluded that although CT produces some improvement in survival the results are not comparable to those seen in malignant teratoma and that radiotherapy should remain a major treatment modality.

### Vitamin D3: Phase I study in low-grade non-Hodgkin's lymphoma

N.L. Gilchrist[1], D. Cunningham[1], R.A. Cowan[2], G.J. Forrest[1], M. Soukop & C.S. McArdle[3]

[1]*Department of Medical Oncology,* [2]*Biochemistry and* [3]*University Department of Surgery, Glasgow Royal Infirmary, UK.*

Vitamin D3 receptors have been demonstrated on established lines of malignant B, T and non-B non-T human lymphocytes but absent on normal resting peripheral B and T lymphocytes. *In vitro* human myeloid leukaemia cell lines demonstrated decreased tumour growth in response to 1αOH Vit D3 or 1α25$(OH)_2$ Vit D3 (Miyaura *et al.* (1981) *Biochem. Biophys. Res. Communi.* **102,** 937). DeLuca & Kodicek have advanced the theory of modification of abnormal lymphocytes through Vit D3 receptors by exogenous 1α25$(OH)_2$ Vit D3.

To examine this hypothesis we prospectively treated 10 patients (6 females and 4 males) with low-grade non-Hodgkin's lymphoma (2 with CLL, 8 with follicular centrocytic NHL) with 1 μg 1αOH Vit D3 daily. All patients were rebiopsied prior to commencement of 1αOH Vit D3 for histological confirmation and Vit D3 receptor assay. All patients were seen at monthly intervals and were assessed clinically, biochemically and haematologically. Two patients with no prior chemotherapy and bulky lymphadenopathy showed impressive clinical responses. Four patients with prior chemotherapy showed stable disease. Four patients with heavy pre-treatment showed progressive disease. No haematological, biochemical or clinical toxicity was observed. The absence of tissue Vit D3 receptors appears to correlate positively with Vit D3 therapy failure.

These results suggest that 1αOH Vit D3 may provide non-toxic therapy for low-grade non-Hodgkin's lymphomas.

**Plasma and CSF methotrexate levels during high dose methotrexate chemotherapy**

N.L. Gilchrist[1], J. Caldwell[2]. I.D. Watson[3], D. Cunningham[1], G.J. Forrest[1] & M. Soukop[1]

[1]*Departments of Medical Oncology,* [2]*Anaesthetics and* [3]*Biochemistry, Glasgow Royal Infirmary. Glasgow, UK.*

Recent intensification of combined modality therapy in high grade non-Hodgkin's lymphoma was initiated to lengthen the duration of remission and to prevent CNS relapse. "High dose" Methotrexate (MTX) has provided a useful addition to such regimes. Drug concentrations of over $10^{-6}$M are recommended for adequate cytotoxicity. (Jolvet *et al.*, 1983, *N. Engl. J. Med.*, 1094).

To assess plasma and CSF MTX levels we studied 4 patients (3 females, 1 male) undergoing combination chemotherapy for high grade NHL. As part of this regime on Day 10 they received i.v. bolus MTX (300 mg m$^{-2}$) followed by a 12 h infusion of MTX (1.2 g m$^{-2}$). Prior to commencement of chemotherapy a lumbar indwelling fine bore CSF catheter was inserted. Paired blood and CSF samples were obtained continuously for 24 h. (Time 0.5, 15, 30, 45, 60, 90 min 2, 3, 4, 5, 6, 8, 12, 15 and 24 h). MTX was measured in plasma and CSF by polarized fluorimetric immunoassay. Creatinine clearances were 108, 109, 91 and 70 ml min$^{-1}$. Plasma results showed initial peak plasma levels of $>10^{-4}$M in all patients. In two patients this dropped to $>10^{-5}$M and was maintained for 12–24 h. But in the further two patients levels dropped below $10^{-5}$M. This was not dependant on renal function. CSF levels were $>10^{-6}$M in those two patients with prolonged and adequate plasma levels, but $<10^{-6}$M in those patients with failure to sustain prolonged levels $>10^{-5}$M.

These results suggest that "CSF sterilization" is not automatic in this "high dose" MTX regime. Plasma levels of MTX should be $>10^{-5}$M for 12 h to ensure adequate cytotoxic levels in the CSF. Care should be taken to ensure constant infusion rate and to give an adequate dose of MTX.

**High dose cyclophosphamide (CTX) and VP16 with autologous bone marrow rescue as late dosage intensification therapy (LDIT) of small cell carcinoma of lung (SCLC)**

D. Cunningham, A. Hutcheon, S.W. Banham, A. Dorward, A.K. Burnett, S. Ahmedzai, P. Tansey, R. Stevenson, M. Soukop, S.B. Kaye & N. Lucie

*West of Scotland Lung Group and Woodend Hospital, Aberdeen, UK.*

Ninety-five patients with SCLC received induction therapy consisting of CTX 1 g m$^{-2}$ Day 1, adriamycin 40 mg m$^{-2}$ Day 1, VP16 100 mg m$^{-2}$ Days 1–3, methotrexate 50 mg m$^{-2}$ and vincristine 2 mg Day 10, 3 weekly. Twenty-two patients showing CR or PR to induction therapy after 3 courses were selected to receive CTX 180 mg kg$^{-1}$, VP16 1 g m$^{-2}$ and mesna 325 mg kg$^{-1}$ as LDIT 4 weeks after completion of induction therapy. Marrow harvested from the patient prior to LDIT was re-infused 36 h later. Prophylactic radiotherapy 4000 cGy was given to the primary site in 10 of the 22 patients 6 weeks after LDIT (see Table below).

Major toxicities were; emesis and myelosuppression 100%, diarrhoea 50%, mucositis 36%, skin rash 23% and haematuria 9%. The survival of patients receiving LDIT was not better than the survival of comparable groups from the 55 patients who received 3 course of induction treatment alone. This pilot study has failed to show any benefit from LDIT in the management of SCLC.

**Table**

| | | *Response to Chemotherapy* | | | | | | *Median* | *Alive in* |
|---|---|---|---|---|---|---|---|---|---|
| | *No. of* | *Induction* | | | *High* | *Dose* | | *Survival* | *Remission* |
| | *patients* | *PR* | *CR* | *None* | *PR* | *PR→CR* | *CR* | *(months)* | *(months)* |
| Limited | 16 | 8 | 8 | 5 | 2 | 1 | 8 | 11 | 3 |
| Extensive | 6 | 3 | 3 | 1 | 1 | 1 | 3 | 10 | — |

**Recovery of haemopoietic stem cells after *in vitro* incubation with cyclophosphamide derivatives used for bone marrow purging in autologous transplantation**

S. Eridani, E. Batten & B. Sawyer

*Department of Haematology, St Thomas' Hospital, London SE 1, UK.*

Autologous bone marrow transplantation is being increasingly tried after intensive chemotherapy in the treatment of neoplastic disorders. Residual tumour cells, either leukaemic or from other cell types, can be removed from freshly collected marrow by different means: cyclophosphamide derivative like 4HC and Asta Z 7557, have also been used for this purpose. Human normal marrow was incubated for 30 min with increasing doses of Asta Z 7557 and then plated to assess granulocytic and erythropoietic colonies (CFU-GM and BFU-E). The results obtained so far show that at dosages up to 5 μg per $2\times10^5$ cells of Asta Z 7557, a dosage commonly used to remove leukaemic cells, there is still a limited amount of colony forming committed cells. A lower recovery rate was seen when the drug was left in the medium for the whole period of culture. In order to test the sensitivity of the primitive haemopoietic progenitors (CFU-S), *in vivo* experiments were also carried out by injecting lethally irradiated mice with syngeneic marrow pre-incubated with dosages of the compound, curative for the L 1210 leukaemic mice. A surviving CFU-S population varying between 5–10% as compared to the controls was found with such dosages.

**Ifosfamide as single agent therapy in children with relapsed solid tumours**

C.R. Pinkerton, J. Pritchard[1], H. Rogers[2] & C. James[2]

[1]*Hospital for Sick Children, Great Ormond Street,* [2]*Department Clinical Pharmacology Guys Hospital, London, UK.*

Long term survival in unresectable and metastatic tumours in childhood remains disappointing despite the use of combination chemotherapy and radiotherapy. The oxazophosphorine, ifosfamide (IF) has been used with some success in adult sarcomas and recently, combined with vincristine, in children (deKraker (1984) *Eur. Paediat. Haematol. Oncol.*, **1**, 47). We have given IF as a single agent to 18 children, aged 2–15 yrs, with relapsed or unresponsive solid tumours. Thirteen had previously received cyclophosphamide (CP)600–1000 mg $m^{-2}$. Five g $m^{-2}$ of IF was administered as a 24 h infusion at 14–21 day intervals. Mesna was given concurrently and for a further 24 h and was highly effective in preventing bladder toxicity. There was no evidence of hepatic or renal toxicity. Myelosuppression was not severe and the count had usually recovered by Day 14. One child had a generalised convulsion after a dose of 7 g $m^{-2}$ which did not recur at the lower dose. One with pre existing renal disease became transiently hypertensive. In most cases vomiting was effectively controlled by high dose dexamethasone. Diagnoses were Ewings sarcoma (3), Wilms tumour (8), rhabdomyosarcoma (3), hepatoblastoma (1) hepatic carcinoma (1), osteosarcoma (1), renal carcinoma (1). There were 2 complete responses (1 Wilms, 1 Ewings) lasting 5 & 9 months respectively. (Localised irradiation was given as consolidation in the latter). There was a partial response in 3 and only a mixed or no response in 12. Plasma IF levels were estimated in 8, mean peak conc. during infusion was 63 μg $ml^{-1}$, elimination $t\frac{1}{2}$, 3.1 h. IF was well tolerated and appeared to be effective in tumours where combination therapy (including CP) had failed. The drug may have a role either as a substitute for CP or as single agent high dose consolidation treatment.

**A method for measuring intestinal damage after intensive chemotherapy and bone marrow transplantation using the absorption of $^{51}$Cr EDTA**

P. Selby, M. Crofts, R.L. Powles & T.J. McElwain

*Department of Medicine, Royal Marsden Hospital, Sutton, Surrey UK.*

Damage to proliferating intestinal epithelia by cytotoxic agents is an important side-effect of cancer treatment and may limit the doses of treatments that can be given. Gut damage may also occur after bone marrow transplantation (BMT) both from the cytotoxic effects of preparation and from graft *versus* host disease (GVHD). Efforts to reduce these effects have been hampered by the absence of reliable and tolerable measurement methods for gut damage in these patients (pts). The absorption of an oral dose of $^{51}$Cr EDTA is a new method for estimating intestinal permeability and we have evaluated it as a measure of gut damage following high dose melphalan (HDM) with autologous bone marrow transplantation or, allogeneic bone marrow transplantation. $^{51}$Cr EDTA (4 MBq) was given as an odourless, tasteless, drink and urinary excretion over 24 h was measured. The test was well tolerated, reliable and had a narrow normal range of up to 2.9% of administered dose in 30 untreated cancer pts. It detected intestinal damage after HDM in 12 pts with a maximum of 8% administered dose after 9 days returning to normal after 15 days. A dose increase of 20 mg m$^{-2}$ produced a significant increase in the peak abnormality indicating the sensitivity of the test. The test also detected damage due to GVHD and 24 h excretion rose to 60% of administered dose in severely affected pts. Clinical abnormalities correlated closely with test results and increased permeability appeared to precede clinical GVHD. The ease, reliability and sensitivity of the test should make it useful in the evaluation of methods designed to reduce gut damage in these clinical situations.

**Transcrecto-sigmoid ultrasonography in the assessment and management of recurrent cervical cancer treated with chemotherapy**

C.A. Meanwell, E.B. Rolfe, G. Blackledge & J.J. Mould

*Queen Elizabeth Medical Centre, Birmingham, UK.*

Fifteen patients with pelvic recurrence of cervical cancer were assessed clinically and radiologically using plain radiographs, abdominal ultrasonography, computerised tomography and transrecto-sigmoid ultrasonography (TRSU). TRSU was performed with patients in the left lateral position using standard transrectal prostate scanners advanced up to 20 cm.

The whole true pelvis was readily demonstrated and pelvic viscera, tumour masses and diseased lymph nodes identified. The procedure was well tolerated by patients.

TRSU showed advantages over clinical examination and abdominal ultrasonography in its ability to document tumour dimensions particularly in the pre-sacral area and in the presence of ascites, dilated bowel and omental disease. TRSU showed advantages over CT in its ability to resolve structures less than 2 cm diameter and to produce images in the coronal or oblique sagittal planes.

TRSU effectively documented the response of tumours to chemotherapy in 6 out of 10 patients subjected to post-treatment examinations.

**A comparative study of a continuous intravenous infusion of high dose metoclopramide with intramuscular chlorpromazine as antiemetic therapy for patients receiving cytotoxic drugs**

D. Cunningham[1], A. Hutcheon[5], M. Soukop[1], S.B. Kaye[4], N.L. Gilchrist[1], G.J. Forrest[1], C.S. McArdle[3] & I.T. Calder[2]

*Departments of [1]Medical Oncology, [2]Pharmacy and [3]Surgery, Glasgow Royal Infirmary, [4]Department of Clinical Oncology, Horselethill, Glasgow and [5]Department of Medical Oncology, Woodend Hospital, Aberdeen, UK.*

Ninety-five patients receiving their first course of cytotoxic therapy entered this double cross-over antiemetic trial comparing a continuous i.v. infusion of high dose metoclopramide (HDM) (5 mg kg$^{-1}$) with i.m. chlorpromazine (25 mg). Seventy-four patients completed the cross-over, 33 of whom received cytotoxic therapy which included cis-platin or cis-platin analogues. In these 33 patients, complete control of vomiting occurred in 27% with HDM and 21% with chlorpromazine. Nausea was less severe with HDM ($P<0.05$) and a significant number of patients preferred HDM as an antiemetic ($P<0.01$). Forty-one patients received cytotoxic therapy without cis-platin with complete control of vomiting in 44% given HDM and 48% given chlorpromazine. In this group no significant differences in the control of nausea or vomiting were observed and there was no overall patient preference.

For patients receiving cytotoxic therapy containing cis-platin HDM was a better antiemetic than chlorpromazine. In other cytotoxic regimes both agents had similar antiemetic efficiency but chlorpromazine is recommended because it is cheaper and easier to administer.

**Experience of angioimmunoblastic lymphadenopathy (AILD) and its possible relationship to T cell lymphoma, in Sheffield**

M.S. Dorreen, N. Rooney[1] & B.W. Hancock

*University Departments of Medicine and [1]Pathology, Royal Hallamshire Hospital, Sheffield S10 2JF, UK.*

Angioimmunoblastic lymphadenopathy (AILD) was diagnosed in 7 previously untreated patients (pts) referred to the Royal Hallamshire Hospital between 1979 and 1983. The dual pathology of AILD + malignant lymphoma (ML) was diagnosed in 2 additional pts. On a subsequent review of all histological material, the diagnosis of AILD was reaffirmed in 5/7 cases. In 3 cases (including the 2 pts. originally diagnosed as AILD + ML) the diagnosis was reclassified as T zone ML, although many of the histological features closely resembled those of AILD. In the case of the 9th pt, a 70 year old male, the diagnosis of lymphoproliferative disease was rejected. The clinical and laboratory features of the 8 pts (5 male, 3 female) retained in the study were indistinguishable, regardless of the pathological diagnosis. The mean age was 65 years (range: 45–75). "B" symptoms were present in 7 pts, of whom 4 presented seriously ill. Other complaints included pruritus (3 pts) and rash (2 pts).

Concurrent medication was of possible aetiological significance in 2 cases. Generalised lymphadenopathy was found in 7 and palpable hepatosplenomegaly, in 6pts. Intrathoracic disease was revealed on the X rays of 5 pts (lymphadenopathy in 4 and parenchymal infiltration in 1 pt). The mean ESR was 45 (range: 4–113). The direct Coomb's test was positive in 3/5 instances. Polyclonal hypergammaglobulinaemia was noted in 6 cases. Two pts observed without therapy remain well at 12 months' follow up. One pt, with T zone ML, died within 4 months of presentation. Three pts were initially treated with prednisolone; 2 have died, one of bronchopneumonia, the other (who presented with AILD) of widespread ML, both within 15 months of first diagnosis; the 3rd pt has undergone histological transformation to a T immunoblastic ML and is receiving treatment for this. Two pts with T zone ML are in complete remission following intensive combination chemotherapy. These results suggest: 1) the accrual of a relatively large number of pts with AILD (4/5 since January 1982) within a single health authority, may indicate a higher UK incidence of AILD than hitherto realised and (2) the clinical and pathological behaviour of AILD suggest a close relationship with certain forms of T cell lymphoma.

**Enzymatic and metabolic profiles in a cachexia model**

M.J. Tisdale[1], R.A. Brennan[1] & K.C.H. Fearon[2]

*[1]CRC Experimental Chemotherapy Group, Department of Pharmacy, University of Aston, Birmingham B4 7ET, [2]Department of Oncology, University of Glasgow, Glasgow GL2 94Y, UK.*

The MAC 16 is a chemically induced transplantable adenocarcinoma of the colon, which produces extensive weight loss in tumour-bearing mice. This weight loss appears to arise from metabolic effects of the tumour, since food intake is not decreased prior to or during weight loss.

We have considered the MAC 16 to be an appropriate model to study the effect of ketosis on the protection of host tissues during the cachectic process. The MAC 16 tumour possesses the three enzymes necessary for ketone body metabolism: 3-oxoacid-CoA transferase, acetoacetyl CoA thiolase and 3-hydroxybutyrate dehydrogenase, although the activity of the first two enzymes is significantly lower than that found in normal colon. There is no significant difference in the enzyme content of non-involved tissues; heart, liver, kidney, brain and colon; between tumour-bearing and non-tumour bearing animals.

Within three weeks after transplantation of the MAC 16 tumour mice weigh an average 5 g less than non-tumour bearing mice, although the average weight of the tumour is only 1 g. During this period blood glucose, lactate, carnitine, acetoacetate and 3-hydroxybutyrate levels in tumour-bearing mice are not significantly different from controls. This suggests a block in the normal metabolic process of ketosis induction host weight loss.

**Comparison of *in vitro* sensitivity in leukaemic cells from blood, bone marrow and lymph node samples**

M.C. Bird, E.D. Gilby & A.G. Bonsanquet

*Department of Clinical Investigation, Royal United Hospital, Combe Park, Bath BA1 3NG, UK.*

A dye-exclusion chemosensitivity assay previously described (Bird *et al.* (1984) *Br. J. Cancer*, **50**, 258) has been used to determine the chemosensitivity of leukaemic cells from simultaneously taken blood (Bl) and bone marrow (BM) samples of 6 patients (3 acute lymphoblastic (ALL), 2 chronic lymphocytic (CLL) and 1 acute myeloid leukaemia (AML). Control viabilities in the assay were similar for the two sites, with a mean of 51% (range 4–86%) for BM, and 59% (22–94%) for Bl samples. Fifteen different drugs have been tested at between one and 6 concentrations per drug (mean of 5 drugs per patient), giving a total of 98 drug sensitivities for comparison. The overall chemosensitivity of BM and Bl leukocytes was very significantly correlated ($r=0.630$; $P<0.001$). Correlation coefficients ($r$) for the three disease categories were 0.881, 0.616 and 0.517 for CLL, AML and ALL patients respectively. Samples were scored as being sensitive ($S$; <30% surviving tumour cells) or resistant ($R$) for each drug concentration tested. Sensitivity was similar for Bl and BM samples in 89 cases (81 $R/R$, 8 $S/S$). Of the remaining 9 data points, 8 were resistant in Bl samples but sensitive in BM. Chemosensitivity was also compared in leukaemic cells from peripheral Bl and lymph node biopsy from a single CLL patient. Control viabilities in the assay were 47% for lymph node and 56% for Bl leukocytes. A total of 22 drug sensitivities from 9 different drugs could be compared, and chemosensitivity was again very significantly correlated ($r=0.748$; $P<0.001$). Determination of sensitivity was in complete agreement with 18 $R/R$ and 4 $S/S$ determinations. The results indicate that samples from any of these three sources could be used equally well to obtain chemosensitivity data for predictive use.

**Possible mechanism of selective toxicity of 1-naphthol to human colonic tumour tissue compared to normal colonic tissue**

M. d'Arcy Doherty, G.D. Wilson & G.M. Cohen

*Toxicology Unit, The School of Pharmacy, University of London, London WC1 1AX, UK.*

The aim of this study was to investigate the possible mechanism(s) of selective toxicity of 1-naphthol to short-term organ cultures of human colonic tumour tissue compared to normal colonic tissue from the same patients (*Biochem. Pharmacol.* (1983) **32,** 2363). At low non-toxic concentrations of 1-naphthol (20 $\mu$M), short-term organ cultures of normal human colon formed predominantly 1-naphthyl sulphate whereas colonic tumour tissue formed more 1-naphthyl-$\beta$-D-glucuronide. At higher concentrations (1 mM), which were selectively toxic to the tumour tissue, conjugation of 1-naphthol to its non-toxic glucuronic acid and sulphate ester conjugates was significantly greater in the normal tissue than the tumour tissue, suggesting an impaired Phase II conjugative metabolism in the tumour tissue. The impaired detoxication pathways resulted in a marked accumulation of unmetabolised 1-naphthol ($42 \pm 12$ nmol mg$^{-1}$ protein) in the tumour tissue compared to that found in the normal tissue ($5 \pm 3$ nmol mg$^{-1}$ protein) following incubation with 1-naphthol (1 mM) for 48 h. The higher levels of 1-naphthol in the tumour tissue may then exert their toxicity by metabolic activation most probably via naphthoquinones. Some evidence for this was obtained by the potentiation of 1-naphthol toxicity by dicoumarol, an inhibitor of DT-diaphorase. In addition, [1-$^{14}$C]-1-naphthol was activated by short-term organ cultures of both normal and tumour tissue to covalently bound products. The level of binding was greater in the tumour tissue suggesting greater conversion to reactive products in this tissue. These results may also be explained by a greater susceptibility of the tumour tissue to oxidative stress.

**A comparison of vitamin A binding in human benign and malignant prostate**

D. Boyd, G.D. Chisholm & F.K. Habib

*Department of Surgery, University Medical School, Teviot Place, Edinburgh EH8 9AG, UK.*

Specific binding sites for [$^3$H] vitamin A alcohol (retinol) have been detected and characterised in the cytosol of human, benign prostate gland. In addition the expression of these sites in the benign and malignant disease has been compared.

Retinol binding sites sedimented to the 2S position on sucrose gradients and were heat/protease inactivated. Competition studies indicated the specificity of binding; unlabelled retinal, retinoic acid and various steroids had little effect on [$^3$H]-retinol bound. Scatchard analysis revealed ligand association to be of high affinity (30–40 nM). The binding characteristics in malignant tissue were found to be similar to those observed in the benign gland.

A cytosol assay was developed to compare retinol binding in benign and malignant gland. The assay was linear with protein concentration over the range 0.1–1.0 mg ml$^{-1}$. Inter- and intra- assay variations were $5.8 \pm 3.8$ and $9.8 \pm 6.9$% respectively. Storage of prostate at $-70$°C for a period of 8 weeks had no adverse effects on the assay.

Retinol binding in benign and malignant prostate was compared and found to be suppressed in the malignant gland (benign $= 4.0 \pm 1.8$; malignant $= 1.7 \pm 1.6$ pmol mg$^{-1}$ protein $\bar{X} \pm s.d.$); these values were shown to be statistically different by the Mann–Whitney U-test. In contrast, no trend was observed between retinol binding and tumour differentiation.

**Immunohistochemical assessment of murine antipancreatic cancer monoclonal antibody DD9E7**

A. Grant[1], E. Heyderman[2], P. Harris[1] & J. Hermon-Taylor[1]

[1]*Department of Surgery, St George's Hospital Medical School and* [2]*Department of Histopathology, St Thomas's Hospital Medical School, London, UK.*

Using a novel immunisation regime, we have generated a number of murine monoclonal antibodies against human pancreatic exocrine cancer (GER) which appear to have sufficient selectivity for pancreatic cancer cells to be of diagnostic and other potential use. Supernatants from these hybridomas were screened on formalin-fixed paraffin-embedded tissue sections. The initial screen was carried out on normal non-neoplastic human pancreas and the original GER pancreatic tumour. Five supernantants stained malignant epithelium in pseudoductules as well as normal pancreatic duct epithelium. DD9E7 produced the most intense staining of malignant epithelium and was further tested against other pancreatic adenocarcinomas and a wide variety of non-neoplastic and malignant tissues in both fixed and frozen tissue sections. The distribution of staining in other tissues, in particular colon, breast and skin carcinomas, suggested it was not recognising CEA or EMA. Consistent staining of polymorphs and macrophages in all sections suggested NAC-like material was being identified but a different pattern of staining was produced to that found with a monoclonal against a shared NCA/CEA determinant.

### Evidence for the lysosomotropic action of an antibody directed drug conjugate

M.C. Garnett, E. Jacobs, M.J. Embleton & R.W. Baldwin

*Cancer Research Campaign Laboratories, University of Nottingham, University Park, Nottingham NG7 2RD, UK.*

The preparation and properties of a methotrexate-human serum albumin-antibody conjugate which retains both antibody binding activity and complete drug cytotoxicity has been previously reported (Garnett *et al.* (1983) *Int. J. Cancer,* **31,** 661). It has been proposed that drug conjugates would be taken up into cells and the drug released by lysosomal enzymes (De Duve *et al.* (1974). *Biochem. Pharmacol.,* **23,** 2495). To test this hypothesis for our conjugate we have assessed the ability of the various inhibitors of lysosomal enzymes to affect the cytotoxicity of both conjugated and free methotrexate to target cells expressing the relevant antigen in a "24 h treatment" assay.

Ammonium chloride, which reduces lysosomal enzyme activity by raising lysosomal pH, reduced the cytotoxicity of both free and conjugated methotrexate. The effect on conjugate was greater, but ammonium chloride also affected cell growth in general. Leupeptin, an inhibitor of both serine and cysteine cathepsins, reduced conjugate cytotoxicity to one fortieth of the uninhibited level with no effect on free methotrexate. E64, a specific inhibitor of cysteine cathepsins, had a similar but smaller effect. Pepstatin A and chymostatin, specific inhibitors of aspartic and serine cathepsins respectively, had no significant effect on the cytotoxicity of the conjugate. These results indicate a lysosomal mechanism of action mediated by cysteine cathepsins, although the contribution of other enzymes cannot be ruled out by these experiments.

### Influence of bestatin, poly I:C, PPD and a pyrimidinone on growth and metastasis of rat mammary carcinoma

M.V. Pimm & R.W. Baldwin

*Cancer Research Campaign Laboratories, University of Nottingham, Nottingham NG7 2RD, UK.*

Bestatin, Poly I:C, PPD and a pyrimidinone (2-amino-3-bromo-6-phenyl-4-pyrimidinone, ABPP, Milas *et al.* (1983) *Clin. Exp. Met.,* **1,** 213) have been tested for therapeutic effectiveness against the metastasising rat mammary carcinoma Sp4.

Bestatin ($5\,mg\,kg^{-1}$ i.p. twice weekly) restricted development of post-surgical lymph node metastases and poly I:C: ($1\,mg\,kg^{-1}$ i.p.) 4, 3, and 2 days before i.v. tumour cell challenge restricted development of pulmonary deposits. Rats pre-immunised with BCG vaccine and showing DTH reactions to PPD, rejected Sp4 cells injected s.c. with PPD. Moreover, i.v. injection of free or liposomally encapsulated PPD restricted the development of post-surgical lymph node metastases and pulmonary tumour deposits. In contrast to the above, ABPP ($250\,mg\,kg^{-1}$ i.p.) 4, 3, and 2 days before i.v. cell injection failed to suppress development of pulmonary deposits. With s.c. tumour challenge ABPP had no influence on tumour take or growth rates. Additional tests were carried out to examine whether ABPP could augment the tumour suppressive effect of DTH reactions to PPD but the effect was abrogated rather then enhanced. To determine whether ABPP influenced the DTH response itself, BCG-immune ABPP treated rats were tested i.d. with graded doses of PPD. The response was significantly reduced in ABPP treated rats compared with controls.

These studies have failed to detect anti-tumour effects of ABPP against the rat mammary carcinoma Sp4, although this tumour is suppressed by Bestatin, poly I:C and PPD. In addition the indication is that ABPP might have some suppressive effect on delayed hypersensitivity reactions.

### Tumour-induced suppression of delayed-type hypersensitivity is independent of cyclophosphamide-sensitive suppressor cells

L.C. McIntosh, L.M. Morrice, Y. Udagawa & A.W. Thomson

*Immunopathology Laboratory, Department of Pathology, University of Aberdeen, Aberdeen Royal Infirmary, Foresterhill, Aberdeen AB9 2ZD, UK.*

The delayed-type hypersensitivity (DTH) response to sheep red blood cells and ovalbumin was markedly reduced in mice bearing the Landschütz ascites carcinoma. Similar effects were obtained by administering cell-free ascitic fluid or serum from tumour-bearing animals together with antigen in the footpad; injection of normal mouse serum had a much less pronounced effect. Treatment with high dose cyclophosphamide prior to immunization did not abolish the effect. Splenic lymphocyte transformation in response to various mitogens was suppressed in tumour-bearing animals while production of a lymphokine (lymphocyte-derived chemotactic factor) remained unimpaired. Therefore, the observed suppression of DTH was not dependent on cyclophosphamide-sensitive T suppressor cells or on the administration of immunosuppressive factor(s) during the induction stage of the response. The tumour may be exerting its immunosuppressive effect by inhibiting lymphocyte proliferation whilst failing to inhibit production of T-cell derived chemotactic factor.

### Stress and breast cancer

T.J. Priestman[1], S.G. Priestman[1] & C. Bradshaw[2]

[1]*Department of Radiotherapy & Oncology, Queen Elizabeth Hospital, Birmingham B15 2TH,* [2]*West Midlands Cancer Research Campaign Clinical Trials Unit, University of Birmingham, UK.*

In order to assess whether exposure to stress was associated with an increased risk of breast cancer, 100 women presenting with carcinoma of the breast completed a standard life events inventory documenting life stresses experienced during the previous three years. The same questionnaire was completed by 100 women presenting with benign breast lumps and 100 healthy controls. Both groups of patients with breast disease also completed the Eysenck personality inventory. There was no difference in the number of stressful life events experienced by the patients with benign and malignant breast lesions and the nature and severity of those stresses encountered were similar for both groups. The personality indices were also the same for both groups. The controls, however, recorded singnificantly higher levels of stress exposure than the patients with breast disease. On the basis of these results there is no evidence to support the hypothesis that stress predisposes to breast cancer development or presentation.

### Viral infections in patients receiving adjuvant chemotherapy for breast cancer

E.H. Dykes[1], R.G. Somerville[2]. D.C. Smith[3] & C.S. McArdle[1]

*Department of Surgery,* [1]*Royal and* [3]*Victoria Infirmaries,* [2]*Department of Virology, Belvidere Hospital, Glasgow, UK.*

Viral infections produce significant morbidity and mortality in immuno-suppressed transplant patients (Ho (1977) *Arch. Virol.*, **5**, 1). Adjuvant chemotherapy is also known to have immunosuppressive effects. The aim of this study was to determine the incidence of viral infection in patients receiving cytotoxic chemotherapy following mastectomy for breast cancer.

One hundred and twenty-four patients attending a breast clinic were studied. Sixty-four patients were currently receiving cytotoxic chemotherapy (CMF), the remaining patients acting as controls. Blood samples were taken at 3 monthly intervals and standard screens for antibodies to respiratory viruses performed. A 4-fold increase in antibody titre between two consecutive samples was considered evidence of recent infection.

Patients receiving CMF had 123 infections during 444 (1 per 3.6) patient months of study. In controls 63 infections occurred during 495 (1 per 7.8) patient months ($P<0.05$). The common viruses encountered were herpes simplex (26%), influenza A (17%) and para influenza (13%). Less common viruses encountered in the CMF patients included RSV (13%), CMV (8%), *M. pneumoniae* (6%).

These results show that patients receiving cytotoxic chemotherapy are at high risk of viral infection and such infections may add to morbidity during treatment.

### Interim results of treatment of breast cancer with breast conservation for all patients

A.S. Bulman, R.H. Phillips & H. Ellis

*Departments of Surgery and Radiotherapy, Westminister Hospital, London SW1P 2AP, UK.*

Randomised studies of mastectomy versus breast conservation are proving difficult for doctors and patients in the United Kingdom to accept, and criteria for selecting patients for one treatment or the other are not agreed. We have therefore adopted a policy of primary treatment with breast conservation for all female patients presenting to this Unit with carcinoma of the breast since March 1979. One hundred and fifty-nine patients had local excision of the tumour with radical radiotherapy up to December 1982. At 1–4 years life tables have indicated overall and recurrence-free survival rates comparable to those expected after mastectomy (Cancer Research Campaign – Kings/Cambridge Trial) – for early breast cancer. Cancer Research Campaign Working Party. *Lancet* (1980), **ii,** 55. Radical *versus* modified radical mastectomy for breast cancer. Turner, Swindell, Bell *et al.* (1981) *Ann. R. Coll. Surg. Engl.* **63,** 239. Local recurrence has occurred in 4/43 patients presenting with Stage I disease, 9/105 Stage II and 1/11 Stage III. Only 4 patients have required mastectomy to control breast recurrence, and of the 9 who have died, only 2 did so with local disease.

**Phase II study of CB3717 in advanced breast cancer**

V. Macaulay, A.H. Calvert, & I.E. Smith

*Medical Breast Unit, Royal Marsden Hospital, Fulham Road, London, UK.*

CB3717 is a quinazoline-based folate analogue which acts by tight-binding inhibition of thymidylate synthetase. Based on phase I data, a phase II study has been started in advanced breast cancer. CB3717 was given at 400 mg m$^{-2}$ q21 with dose reduction for impaired glomerular filtration rate (GFR) or elevated alkaline phosphatase (AP). Seventeen patients have been entered and so far 11 are evaluable, of whom 8 had previous combination chemotherapy. Two patients achieved PR for 13 and 20 weeks, and there were 2 minor responses (<50% reduction of 10+ and 17 weeks. Three patients had SD and 4 had PD, including one mixed response. Response rates by metastatic site are: soft tissue 3/11, visceral 3/11, osseous 1/4. Median time to progression is 9 weeks (4+ −21). Nephrotoxicity was dose-limiting with 20–50% fall in GFR in 71% and >50% in 14%. No patient required dialysis and there is early evidence of reversibility in one patient. Elevation of liver function tests (LFTs) was seen in 92%, affecting ALT ($\leqslant$3 × normal in 50% of patients, >3 × normal in 33%) but also AP (75% and 0%) and Gamma GT (58% and 17%). This was associated with malaise, which occurred 3–10 days after treatment, in 46% of patients. Prednisolone cover relieved malaise in all cases. LFTs spontaneously improved in 4 of 7 patients given more than 2 courses. Other toxicities included nausea/vomiting (19%) and conjunctivitis (12.5%); myelotoxicity was not seen (median nadir Hb. 9.9 g dl$^{-1}$, WBC 6.4, platelets 247 × 10$^{9}$ l$^{-1}$. These preliminary results confirm that CB3717 is active in advanced breast cancer, with minimal myelosuppression but significant renal and symptomatic hepatic toxicity. Responses are brief in these heavily pre-treated patients; the drug may be more effective as first-line chemotherapy.

**Mitomycin C and vinblastine in the treatment of advanced breast cancer**

J.A. Radford & R.D. Rubens

*Imperial Cancer Research Fund Breast Cancer Unit, Guy's Hospital, London, SE1 9RT, UK.*

Response of advanced breast cancer to secondary chemotherapy is poor, but response rate of 40% has been reported (Konits *et al.* (1981), *Cancer.* **48,** 1295). We have treated 35 patients with advanced breast cancer progressive after at least one prior chemotherapy regimen, with mitomycin C 12 mg m$^{-2}$ (max. 20 mg) and vinblastine 6 mg m$^{-2}$ (max. 10 mg) i.v. every 3 weeks. Twenty-six had received prior endocrine treatment and all had received prior chemotherapy (21 one and 14 two or more regimens, including Adriamycin, mitoxantrone, or combined cyclophosphamide + methotrexate + 5-fluorouracil). Five patients had received interferon and 13 radiotherapy to metastases. Twenty-three patients are evaluable after 2 or more courses. Seven (32.8%) have responded (1 CR, 6 PR). Response at sites were: breast 6/17, skin 5/18, lymphatic 3/14, bone 0/10, liver 0/4, lung 0/7, pleura 1/2. Toxicity was mild. Eight had nausea or vomiting < WHO grade 2, one had alopecia, WHO grade 1 and one had diarrhoea, WHO grade 1. Median lowest recorded Day 22 white blood cell count was 3.75 × 10$^{9}$ l$^{-1}$, platelets 172 × 10$^{9}$ l$^{-1}$ and percent projected doses given were mitomycin C 86.0% and vinblastine 87%.

Mitomycin C + vinblastine is active as second or third line chemotherapy for advanced breast cancer with minimal toxicity. Accrual to this study continues.

**Bone scintigraphy in breast cancer: A nine year follow-up**

I.H. Kunkler, M.V. Merrick and A. Roger

*Departments of Clinical Oncology and Nuclear Medicine, Western General Hospital, Edinburgh, UK.*

The results of bone scintigraphy (BS) in 465 women with histologically proven breast cancer were correlated with tumour size, nodal status (NS), clinical course and survival. Median follow-up periods were 26, 26 and 20 months respectively for BS positive (+), BS negative (−) and BS benign/equivocal (B/E). The maximum follow-up of the whole group was 9 years. (See Table below.)

**Table**

| *Staging* | $T_{0-1}$ | | | | $T_2$ | | | | $T_3$ | | | | $T_4$ | | | |
|---|---|---|---|---|---|---|---|---|---|---|---|---|---|---|---|---|
| BS | *N*+ | | *N*− | | *N*+ | | *N*− | | *N*+ | | *N*− | | *N*+ | | *N*− | |
| | *No* | (%) | *No* | (%) | *No* | (%) | *No* | (%) | *No* | (%) | *No* | (%) | *No* | (%) | *No* | (%) |
| BS (−) | 20 | (77) | 57 | (86) | 85 | (72) | 81 | (72.3) | 20 | (50) | 21 | (84) | 33 | (56) | 11 | (58) |
| BS(+) | 2 | (8) | 1 | (2) | 10 | (8.5) | 7 | (6.3) | 7 | (17.5) | 2 | (8) | 12 | (20) | 3 | (16) |
| BS (B/E) | 4 | (15) | 8 | (12) | 23 | (19.5) | 24 | (21.4) | 13 | (32.5) | 2 | (8) | 14 | (24) | 5 | (26) |

The average incidence of BS(+) was 10.1%. Skeletal metastases (SM) were eventually confirmed radiologically or at post-mortem in 17.6% of the patients but had been identified by BS at presentation in only 9.5%. 13.6% of BS(+) failed to develop confirmatory evidence of metastases during follow-up. No correlation was found between histological NS and BS(+) ($P=0.79$). No significant difference was detected between BS(−) and BS(B/E) in the subsequent development of SM ($P=0.5$). The actuarial survival over 9 years was significantly shorter for BS(+) than B(−) or BS(B/E) ($P=0.03$). There is no evidence that routine BS affects the management of newly diagnosed breast cancer. Unless an algorithm can be developed which requires this information, routine BS is not justified. It should be reserved for patients with clinical suspicion of metastases and for clinical trials.

### Tamoxifen as primary therapy for elderly women with breast cancer

S.G. Allan[1], U. Chetty[2], A.P.M. Forrest[2], A. Rodger[1], J.F. Smyth[1] & R.C.F. Leonard[1]

[1]*University Department of Clinical Oncology, Western General Hospital,* [2]*University Department of Clinical Surgery, Royal Infirmary, Edinburgh, UK.*

Since 1977, 105 women, mean age 76.3, with histologically confirmed breast carcinoma of any stage have received primary therapy with tamoxifen 10 mg tds indefinitely or until tumour progression (range 5–55 months). Thirty-eight patients, 36%, had other major system disorders e.g. vascular disease, dementia, anthropathy.

One hundred patients are evaluable for response with known oestrogen receptor (ER) status in 37 (median ER 300 fmol mg$^{-1}$ cytosol prot). The 70% response rate (43% CR) in the known ER positive group is not dissimilar to the overall response rate (39% CR). Median time to best response was 15.5 weeks (range 6–135). Median duration of tamoxifen therapy is 23 months (range 5–48) for CR patients, 18 months for PR (range 6–55) and 15 months for the no change group. Twelve of the 68 responders (2 CR, 10 PR) have relapsed giving a 19 month median response duration (mean 24 months, range 9–55 months). Ten patients had progressive disease despite tamoxifen. Locally advanced disease appeared to influence response with only a 53% response rate in the "T4" group.

Side effects to tamoxifen were absent in 67% with dry mouth 13%, transient nausea 10%, vomiting 4%, fatigue 10%, vaginal dysaesthesia 2% and vaginal discharge 2%. Only 5/14 deaths were due to carcinoma, these being in non-responders.

### DNA interstrand cross-linking and drug sensitivity to cis-diamminedichloroplatinum II (CDDP) in human ovarian cancer cell lines *in vitro*

B.G. Ward & B.T. Hill

*Laboratory of Cellular Chemotherapy, Imperial Cancer Research Fund, London, WC2A 3PX, UK.*

CDDP has proven to be effective agent against human epithelial ovarian cancer. No study of the mode of action of the drug has yet been reported using a human tumour model system.

Four human epithelial ovarian cancer cell lines have been assayed for sensitivity to CDDP *in vitro* by the soft agar colony-forming assay of Courtenay. (Courtenay & Mills (1978) *Br. J. Cancer*, **37**, 261). Assays were performed after the cells were incubated for 1 or 24 h with CDDP and the results expressed as the concentration of drug required to limit colony formation to 50% of control untreated cells ($ID_{50}$). In the 4 lines, mean $ID_{50}$ values ranged from 280 ng ml$^{-1}$ to 4.3 $\mu$g ml$^{-1}$ (1 h exposure) and from 26 ng ml$^{-1}$ to 680 ng ml$^{-1}$ (24 h exposure).

Following a 1 h incubation with CDDP at various concentrations single cell suspensions were exposed to a known X-ray dose to induce DNA single strand breaks. DNA interstrand cross-linking was then measured against these by the alkaline denaturation-renaturation method of Jolley and Ormerod. (Jolly & Ormerod (1973) *Biochim Biophys Acta.*, **308**, 242). Results are expressed in rad equivalents. This technique permitted quantitative comparative studies with these 4 cell lines. In each case, following a 1 h exposure to CDDP, cross-links formed linearly with increasing dose, reaching a maximum 4–8 h after drug removal. Mean interstrand cross-link formation ranged from $88 \pm 9$ to $233 \pm 26$ rad equivalents at 4 hrs after a 1 h exposure to 40 $\mu$g ml$^{-1}$ CDDP and these levels reflected the relative drug sensitivities of the cell lines, with one exception. These results suggest that DNA interstrand cross-links may be associated with the cytotoxicity of CDDP but do not support a causal relationship.

### Verapamil enhances the sensitivity to adriamycin and VP16-213 of human lung cancer *in vitro*

C.A. Fetherston, S. Merry, S.B. Kaye & R.I. Freshney

*Department of Clinical Oncology, University of Glasgow G12 9LX, UK.*

In some experimental tumour models resistance to adriamycin and VP16 has been attributed to enhanced cellular drug efflux, and sensitivity to adriamycin has been increased using calcium channel blockers, including verapamil. In this study, the $ID_{50}$ values of 6 continuous human non-small cell lung cancer cell lines to 7 drugs (adriamycin, actinomycin D, VP16-213, vincristine, 5-FU, mitomycin C and melphalan) were determined. Cells were seeded onto microtitre plates and, after 72 h, were exposed to drug for a further 72 h followed by a recovery period of 120 h. Cell number at the end was determined by tritiated-leucine incorporation into the trichloroacetic acid insoluble fraction (Freshney *et al.* (1975) *Br. J. Cancer,* **31,** 89). Of 3 cell lines resistant to adriamycin (L-DAN, WIL, A549) a non-cytotoxic concentration of verapamil (6.6 μM) caused a 6–10 fold decrease in $ID_{50}$ in 2 of the lines. In H125 (sensitive) 6.6 μM verapamil had no effect. With VP-16, 6.6 μM verapamil caused a 3–6 fold decrease in $ID_{50}$ in 3 cell lines resistant to the drug (WIL, A549, H125), although in another VP16 resistant line (SK-MES) verapamil had no effect. Four of the cell lines used were also resistant to vincristine, but 6.6 μM verapamil only caused a 2-fold reduction in $ID_{50}$ of one cell line (WIL). These results demonstrate that verapamil is able to increase the sensitivity of some, but not all, human lung carcinoma cell lines, and may indicate a potential role for verapamil in circumventing clinically observed drug resistance. Since verapamil had an effect on only some of the cell lines, different mechanisms of resistance may be in operation. Studies on membrane transport as one possible mechanism are in progress.

### Factors affecting drug release from adriamycin-loaded protein microspheres

N. Willmott[1], J. Cummings[2], J.F.B. Stuart[1] & A.T. Florence[1]

[1]*Department of Pharmacy, University of Strathclyde and* [2]*Department of Clinical Oncology, University of Glasgow, UK.*

Microspherical drug delivery systems hold out the possibility of increasing the specificity of cytotoxic anti-cancer agents by (a) tissue localisation through capillary blockade of particular organs with particles of requisite size and (b) control of drug release from carrier. In order to examine the latter point a system was developed that involved immobilising drug-containing microspheres on a glass wool column subjected to aqueous buffer at a constant flow rate, and analysis of the eluate for adriamycin (Adx).

Time taken for elution of 50% of Adx ($T_{50}$), measured by total fluorescence and high pressure liquid chromatography, was compared with regard to (a) protein (albumin, haemoglobin) used as the microsphere matrix, (b) concentration of cross-linking agent (glutaraldehyde) used in microsphere formation and (c) microsphere size. It was found that for Adx-loaded microspheres prepared using 1% glutaraldehyde, albumin ($T_{50}$ = 3.5 h) gave more protracted drug elution than haemoglobin or free drug ($T_{50}$ = 2.6 h and 2.2 h). Moreover, for albumin microspheres, increasing glutaraldehyde concentration and particle size both decreased elution rates, although the maximum $T_{50}$ was still only 5 h.

Total drug content was measured after digestion of microspheres with trypsin and in this case total fluorescence measurements were a reliable measure of Adx content only under certain conditions; namely, using albumin as matrix protein and $<1\%$ glutaraldehyde. Under such conditions it was observed that not all drug was released *in vitro.*

These data are consistent with *in vivo* results showing an initial, relatively rapid release of a proportion of drug from intact microspheres, with the remainder only becoming available when the structural integrity of the particles is compromised.

### A study of adriamycin resistance using human ovarian tumour cell lines

E.M. Gibby, O. Boyse & B.T. Hill

*Laboratory of Cellular Chemotherapy, Imperial Cancer Research Fund, Lincoln's Inn Fields, London, WC2A 3PX, UK.*

Ovarian carcinoma is the fifth most common cause of death from cancer amongst women in the United Kingdom. A problem in treating this disease is the development of resistance to drugs used as a second line agents. This is particularly marked for adriamycin (ADR) so we are using experimental models of human ovarian cancer to investigate response to ADR *in vitro.* Three human tumour continuous cell lines, established directly from ovarian cancer biopsy specimens have been studied. There is an inherent heterogeneity of response to a 24 h exposure to ADR using the Courtenay clonogenic assay with $IC_{50}$ values ranging from 10–35 ng ml$^{-1}$. Duration of drug exposure is an important determinant of drug-induced cytotoxicity. In one cell line (SK-OV-3) a comparison of $IC_{50}$ values for 1 h and 24 h exposures to ADR indicates a significantly higher "CxT" value for the longer exposure. The different responses to ADR shown by the 3 cell lines cannot be explained by the 5-fold variation in net drug uptake of this drug by the cells, measured fluorimetrically.

To study the development of resistance to ADR *in vitro,* a series of sublines of the SK-OV-3 cell line have been produced by intermittent, repeated exposures to a range of clinically-achievable plasma drug levels. The subline treated with the highest concentration ($6 \times 200$ ng ml$^{-1}$) exhibits an approximate 10-fold order of resistance compared with the parent cells. This sub-line exhibits no cross-resistance to 4′-deoxy-ADR; is sensitive to a lower concentration range of this 4′-deoxy derivative compared with ADR; shows no defect in ADR uptake measured fluorimetrically; and the resistance to ADR is not overcome by verapamil. Alternative mechanisms implicated in this expression of drug resistance are now being investigated.

### Resistance to antimicrotubular drugs in CHO cells

J.R. Warr, F. Brewer, K. Adams & M. Anderson

*Biology Department, University of York, York YO1 5DD, UK.*

Several aspects of resistance to antimicrotubular anticancer agents in Chinese hamster ovary (CHO) cells have been studied. The drugs studied include vincristine, vindesine and taxol. Mutants have been selected either by a single challenge to a high drug concentration or by prolonged exposure to gradually increasing drug concentrations over a period of months.

Stable mutants selected by the former method show several interesting features in their cross-resistance patterns. We have previously reported that some vincristine resistant strains are hypersensitive to the microtubule stabilising drug taxol (Warr *et al.* (1984) *Cell. Biol. Int. Rep.*, **8**, 591) and we have now analysed large numbers of independently isolated vincristine or taxol resistant mutants for their pattern of reciprocal cross-resistance or sensitivity. There is not a consistent pattern of reciprocal sensitivity. Genetical analysis (by complementation studies in somatic cell hybrids), analysis of the cross-resistance patterns to other classes of antitumour drugs and other phenotypic differences between resistant cell lines suggest that mutations in several different genes may be involved in resistance to vinca alkaloids in CHO cells.

Selection for resistance to vincristine by prolonged exposure to the drug has produced cell lines with around a hundred fold increase in vincristine resistance. Resistance is unstable during prolonged culture in drug-free medium and the cell lines are shown to have reduced vincristine uptake.

### Derivation and characterisation of a multidrug resistant line of human small cell lung cancer cells

P. Twentyman, N. Fox & N. Bleehen

*MRC Clinical Oncology and Radiotherapeutics Unit, Hills Road, Cambridge, UK.*

There are numerous reports in the literature of the production of drug-resistant cell lines by growth in increasing concentrations of various drugs. The great majority of such reports describe cell lines of mouse or hamster origin and those which describe human cells are almost exclusively confined to leukaemias and lymphomas. Although we have recently derived, without difficulty, adriamycin (ADM) resistant sublines from 2 murine solid tumour lines (EMT6 and RIF-1), it has proved extremely difficult to obtain such a subline from a range of human lung cancer cell lines of various types. We have now, however, isolated an ADM-resistant subline (LX) of the small cell lung cancer line NCI-H69 (originally provided by Dr. D. Carney). The subline LX was obtained by serial growth in the presence and absence of ADM over a period of 9 months. The sensitivity to ADM of LX is much lower than the sensitivity of 14 other human lung cancer cell lines studied. Subline LX is also very resistant to colchicine and vincristine but similar to the parent line in sensitivity to melphalan, CCNU, bleomycin and aclacinomycin A. Intracellular ADM concentrations after acute exposure are reduced in LX cells compared with the parent cells. Resistance to ADM of LX is lost only slowly during growth in the absence of the drug. Continuing studies are concerned with an examination of membrane glycoproteins in subline LX and determination of cellular pharmacokinetics for a range of anthracyclines.

### Whole body hyperthermia – effects on the pharmacokinetics of two nitroimidazole radiosensitizers SR 2508 and Ro 03-8799 in mice

M.I. Walton, N.M. Bleehen & P. Workman

*MRC and University Department of Clinical Oncology, Hills Road., Cambridge, CB2 2QH, UK.*

Whole body hyperthermia (WBH) is currently being evaluated in combination with chemotherapy, through there have been very few detailed studies on the effects of WBH on drug pharmacokinetics. Nitroimidazole radiosensitizers show enhanced tumour cytotoxicity and radiosensitization with heat *in vitro*, and we have investigated the effects of WBH in mice on the pharmacokinetics of two radiosensitizers undergoing clinical trial. These are SR 2508, a hydrophilic neutral nitroimidazole, and Ro 03-8799, a basic lipophilic nitroimidazole. Drugs were given i.v. to C3H mice bearing KHT tumours, 10 min before WBH in an incubator (core temp, $41 \pm 0.50$°C for 45 min). Both compounds were given at 60% heated $LD_{50}$ dose, i.e. 3 mmol $kg^{-1}$ SR 2508 and 0.69 mmol $kg^{-1}$ Ro 03-8799. Plasma, tumour and brain drug concentrations were measured in heated and control mice using HPLC. Heat increased the acute toxicity of SR 2508 and Ro 03-8799 5-fold and 3-fold respectively. WBH significantly prolonged the terminal half-life ($t_{\frac{1}{2}}$) of SR 2508 from $83 \pm 5$ min to $121 \pm 2$ min (95% conf, $P<0.001$). It also increased Ro 03-8799 $t_{\frac{1}{2}}$ by 26% from $23 \pm 2$ min to $29 \pm 2$ min (95% conf, $P<0.001$). Plasma clearance was reduced in heated mice from 1.1 to 0.7 ml $hr^{-1} g^{-1}$ for SR 2508 and from 3.89 to 3.19 ml $h^{-1} g^{-1}$ for Ro 03-8799. WBH greatly inhibited glomerular filtration specifically during the heating period, as measured by $Cr^{51}$ EDTA clearance. Ro 03-8799 tissue/plasma ratios were decreased by WBH, e.g. at 60 min tumour/plasma ratios were reduced by 44% from 2.5 to 1.4. WBH did not alter SR 2508 brain/plasma ratios but slightly reduced tumour/plasma ratios, e.g. from 1.22 to 1.12 at 2 h. In conclusion, WBH profoundly affects plasma pharmacokinetics and renal clearance of Ro 03-8799 and particularly SR 2508. It also reduces Ro 03-8799 tumour/plasma and brain/plasma ratios at later times.

### Induction of differentiation of HL60 leukaemic cells *in vitro* by analogues of N-methylformamide and the relationship with antitumour activity and toxicity *in vivo*

S.P. Langdon & J.A. Hickman

*CRC Experimental Chemotherapy Group, University of Aston, Birmingham, UK.*

N-Methylformamide (NMF), a polar solvent, is in phase 2 clinical trial. Dexter *et al.* have suggested that its antitumour action might be related to its ability to induce differentiation in tumour cells (*Cancer Res.* (1982) **42,** 5018). We have studied the ability of a series of analogues of NMF to induce differentiation in HL60 promyelocytic cells *in vitro* and have related this to their toxicity and antitumour activity *in vivo*. The general formula of these analogues was $R^3CONR^1R^2$ where $R^1 = H$, Me, Et; $R^2 = H$, Me, and $R^3 = H$, Me, $NH_2$ NHMe or $NMe_2$. Differentiation was assessed by the ability of cells to phagocytose yeast and reduce nitroblue tetrazolium. Each of the analogues tested was able to induce differentiation *in vitro* over a narrow concentration range. A simple inverse relationship appears to exist between the optimal inducing concentration, the cytotoxic concentration *in vitro* and the molecular weight of the compound. This suggest that the effects of *in vitro* differentiation and cytotoxicity may be mediated in a relatively non-specific manner. In contrast to the *in vitro* differentiation results, only NMF was active against murine tumours *in vivo*. The methylated acetamides and ureas were inactive *in vivo*, despite being less toxic than NMF and being more potent inducers of differentiation. While 150 mM NMF was required to induce differentiation *in vitro*, 7 mM is the maximum plasma concentration that may be maintained in mice. These results suggest that unless NMF is metabolized *in vivo* to a more potent inducing species, it seems unlikely that its antitumour action and its ability to induce differentiation are related. Additionally, the methylated acetamides and ureas appear to be more promising candidates for the induction of terminal differentiation of human tumour cells *in vivo* than NMF.

### Treatment of two transplantable tumours with the putative anti-angiogenic combination of heparin plus cortisone

M. Penhaligon & R.S. Camplejohn

*Richard Dimbleby Department of Cancer Research, St Thomas's Hospital Medical School, London SE1 7EH, UK.*

Folkman *et al.* (1983) *Science,* **221,** 719 reported that heparin combined with cortisone caused regression and complete cure of a number of transplantable tumour types. Some of these tumour types, e.g. 3LL and B16, grow rapidly and metastasize and are not usually curable by chemotherapy. Folkman's work suggested that these stimulating results involved an inhibition of tumour angiogenesis. However, the efficacy of this therapy was dependent on the source of heparin and the most potent, Panheparin (Abbott) is no longer being manufactured. We have made an initial study of heparins from five different manufacturers (Weddel, Monparin; Sigma; Leo; Boots; Organon, Diosynth) differing in source (pig/cow, lung/intestine) and in degree of purity. C3H/He mice bearing either C3H mouse mammary adenocarcinomas or RIF-1 fibrosarcomas of approximately 180 $mm^3$ volume were treated with a combination of heparin (500 anticoagulation units $ml^{-1}$ drinking water) plus cortisone (250 mg $kg^{-1} day^{-1}$ s.c. tapering to 37 $mg^{-1} day^{-1}$ or a constant dose of 75 mg $kg^{-1} day^{-1}$) these being the drug doses and schedules found effective in Folkman's studies. RIF-1 tumours shrank to approximately half the volume at the start of therapy after only 3 days of treatment; mammary tumours took longer to respond, not reaching half the starting volume until after 11 days of treatment. However, response to combined heparin and cortisone therapy was in fact no different from the response to cortisone used alone and in both tumour types the response was transient and tumours eventually regrew. Also, cortisone treatment was extremely toxic to these animals and experiments had to be terminated after about 3 weeks of therapy. These results differ from those of Folkman *et al.* who found no antitumour effect using cortisone alone and experienced none of the early toxic effects seen in this study.

**Nucleotide "prodrugs" of 6-mercaptopurine and cytosine arabinoside**

D.M. Tidd, H.P. Johnston, P. Hawley, S. White & I. Gibson

*School of Biological Sciences, University of East Anglia, Norwich, NR4 7TJ, UK.*

Using 6-mercaptopurine (MP) as a model system we have demonstrated that the efficacy of anti-metabolite nucleotide "prodrugs" designed to circumvent drug resistance may be limited by their hydrophilic character and by the degradative activity of serum phosphodiesterases. Bis(thioinosine)-5′, 5′′′-phosphate [(bisMPR)P], an MP nucleotide prodrug originally synthesized by Montgomery *et al.* (1963 *Nature*, **199**, 769); was cytotoxic to our thiopurine-resistant L1210/MPR cell line in culture; however, the extent of its effects was limited by its intrinsically slow action and its relatively rapid extracellular degradation. Esterification of the sugar hydroxyls with butyric acid enhanced the rate of action of bis(MRP)P and afforded considerable protection against breakdown by serum enzymes. Both bis(MRP)P and the lipophilic butyryl derivative, bis(dibut.MPR)P induced progressive inhibitions of incorporation of radiolabelled precursors into cellular RNA, DNA and protein. Bis(dibut.MPR)P was more cytotoxic than bis(MPR)P against L1210/MPR cells, and also against thiopurine-resistant Chinese hamster CH/TG cells which were equally insensitive to MP riboside and bis(MPR)P itself. The properties of the MP derivatives were compared with those of analogous compounds of cytosine arabinoside.

**Cerebral photosensitization by haematoporphyrin derivative (HPD)**

M.C. Berenbaum[1], G.W. Hall[1] & A.D. Hoyes[2]

[1]*Department of Experimental Pathology,*
[2]*Department of Anatomy, St Mary's Hospital Medical School, London, W2 1PG, UK.*

Photodynamic therapy (systemic administration of tumour-localising porphyrin followed by exposure of the tumour to light) may be a useful treatment for malignant brain tumours as (i) porphyrins are excluded from most of the brain by the blood-brain barrier (BBB) but not from tumours, (ii) the brain is relatively translucent to light of the appropriate wave-lengths, (iii) brain tumours rarely metastasize and so, in principle, the whole tumour can be illuminated, (iv) the results of other treatments are poor.

However, we find that injection of HPD in rodents produces a long-lasting ($>3$ months) photosensitization of normal brain, exposure of the cranium to light causing a rapid and usually fatal cerebral oedema. Surviving animals show necrosis of illuminated areas of brain. The possibilities are either (i) HPD, contrary to current belief, does pass the BBB or (ii) HPD is retained for a remarkably long time on the vascular side of the BBB. Our evidence suggests the latter, viz (i) the oedema is of vasogenic origin, (ii) the brain is immediately sensitized by an injection of protein-bound HPD, (iii) conventional histology shows early endothelial cell necrosis, (iv) electron microscopy shows opening of endothelial tight junctions. These findings may be relevant to the mechanisms by which photodynamic therapy damages tumours.

The organisers acknowledge the generous support of the cancer research campaign and the Imperial Cancer Research Fund.